# Quantitative Genetics of the Aging of Reproductive Traits in the Houbara Bustard

**DOI:** 10.1371/journal.pone.0133140

**Published:** 2015-07-28

**Authors:** Stéphane Chantepie, Alexandre Robert, Gabriele Sorci, Yves Hingrat, Anne Charmantier, Gwénaëlle Leveque, Frédéric Lacroix, Céline Teplitsky

**Affiliations:** 1 UMR 7204 MNHN-CNRS-UPMC Centre d’Ecologie et des Sciences de la Conservation, Muséum National d'Histoire Naturelle, Paris, France; 2 UMR CNRS/uB 6282 Biogéosciences, Université de Bourgogne, Dijon, France; 3 Reneco for Wildlife Preservation, Abu Dhabi, United Arab Emirates; 4 UMR 5175 CEFE-CNRS Centre d’Ecologie Fonctionnelle et Evolutive, Montpellier, France; 5 Emirates Center for Wildlife Propagation, Missour, Morocco; University of Arkansas, UNITED STATES

## Abstract

Do all traits within an organism age for the same reason? Evolutionary theories of aging share a common assumption: the strength of natural selection declines with age. A corollary is that additive genetic variance should increase with age. However, not all senescent traits display such increases suggesting that other mechanisms may be at play. Using longitudinal data collected from more than 5400 houbara bustards (*Chlamydotis undulata*) with an exhaustive recorded pedigree, we investigated the genetics of aging in one female reproductive trait (egg production) and three male reproductive traits (courtship display rate, ejaculate size and sperm viability), that display senescence at the phenotypic level. Animal models revealed an increase in additive genetic variance with age for courtship display rate and egg production but an unexpected absence of increased additive genetic variance for ejaculate size and no additive genetic variance for sperm viability. Our results suggest that the mechanisms behind the senescence of some traits are linked with a change in genetic expression, whereas for some other traits, aging may result from the constraints associated with physiological wear and tear on the organism throughout the life of the individual.

## Introduction

Aging is defined as a progressive decline in the age-specific fitness components of an organism due to internal physiological degeneration [1,2]. From an evolutionary perspective, senescence arises from the declining force of natural selection with age because the cumulative risk of extrinsic mortality increases with age [3–5]. Because population genetics theory predicts that directional or stabilizing selection will reduce additive genetic variance [6], a corollary is that the relaxation of the force of selection at older ages should be associated with an increase in additive genetic variance for senescing traits. This prediction is common to both evolutionary theories explaining aging: the 'mutation accumulation theory' (MA) that assumes the accumulation of alleles with late-acting deleterious effects [7], and the 'antagonistic pleiotropy theory' (AP) that assumes selection of alleles with advantageous early life effects but deleterious late-acting effects [3]. In fact, both theories predict an increase in additive genetic variance after the age of primiparity [8,9].

Empirical studies have provided mixed results regarding changes in additive genetic variance at older ages. Historically, the genetic theories of aging have been largely supported in diverse laboratory model species, including invertebrates, vertebrates and plants (e.g., *Drosophila melanogaster*, [10,11] mouse *Mus musculus* [12] and thale cress *Arabidopsis thaliana* [13]). More recently, studies from wild populations have reported age-related genetic patterns supporting both theories in vertebrates and plants (e.g., Mute swan *Cygnus olor* [14,15], red deer *Cervus elaphus* [16,17], Soay sheep *Ovis aries* [18], Bighorn sheep *Ovis Canadensis* [18] and *Silene latifolia* [19]). However, other studies have reported patterns incompatible with the predictions of the genetic theories of aging. Indeed, in *Drosophila*, the additive genetic variance is known to be stable with age for egg production [20], whereas it has repeatedly been shown to decrease for mortality [21–25]. However, the reasons behind this decline are still not fully understood. In birds, additive genetic variance for fitness has been shown to be stable with age [26,27], and the authors of these studies suggested that the reasons for individual variation in senescence rates were mostly ecological rather than genetic. Overall, the discrepancy between these results and theoretical predictions represent a serious challenge for genetic theories of aging. In addition to the lack of increase in additive genetic variance, another discrepancy between these empirical results and the AP and MA theories is the fact that the onset of senescence often occurs later than the age of primiparity [28]. Hence, although MA or AP theories have been empirically validated to some extent, a remaining fundamental question concerns whether other theories should also be invoked to explain aging.

Senescence patterns have usually been measured on single traits, but recent studies have highlighted that within the same organism, different traits can vary in their onsets and rates of phenotypic senescence [29–31]. The variability of senescence rates among traits is consistent with laboratory results suggesting that age-related deterioration of different traits may be independently regulated by different genes [32]. Furthermore, the rate of senescence can also differ between sexes. In numerous studies, senescence began earlier and was faster in males than in females (e.g., [30,31,33,34]), especially in polygynous species [35]. Thus, exploring the genetics of senescence on multiple traits and both sexes appears to be a necessity for drawing general conclusions about senescence.

In this study, we explored the genetic basis of senescence in male and female reproductive traits of the houbara bustard (*Chlamydotis undulata*), a non-domestic and non-laboratory bird species in which phenotypic patterns of senescence have been found in both male and female reproductive parameters. A recent study [36] uncovered very strong patterns of senescence at the phenotypic level in three sperm traits (sperm production, morphology and motility) and only moderate senescence in a male behavioral trait related to reproduction (courtship display rate, see discussion). There is also strong phenotypic senescence in the number of eggs produced by females.

Based on longitudinal individual observations from more than 2400 males and 3000 females ranging from 1 to 23 years old with an exhaustive pedigree record, we quantified the age-related variation of additive genetic variance of one male behavioral sexual trait (courtship display rate, see [37]), one male sperm production trait (ejaculate size), one male sperm performance trait (sperm viability) and one female performance trait (number of eggs produced). Moreover, as negative genetic correlations between early and late expression of trait are expected under AP theory [9], we also estimated the among-ages genetic correlations for all traits. If aging in this species is explained by genetic factors, according to MA and AP evolutionary theories of aging, we expect an increase of additive genetic variance with age from age of primiparity and, under the AP mechanism, a negative genetic correlation between early and late expression of traits.

## Materials and Methods

### Data collection & traits

The north-African houbara bustard *Chlamydotis undulata undulata* is a medium-sized endangered bird that inhabits semi-arid areas in North Africa and in the Canary Islands. The sharp decline of the populations, mainly due to over-hunting and habitat degradation, [38] led to the creation, in 1996, of the Emirates Center for Wildlife Propagation (ECWP) based in eastern Morocco. The ECWP captive breeding program aims at increasing population sizes of the endangered houbara bustard throughout its range in North Africa [39]. The data used for this study were collected by the ECWP. The breeding program and captivity conditions were approved by the Moroccan ministry of Agriculture and by a mandated independent veterinarian (mandate number: 534–98). Individuals were fed *ad libitum* and cared for by a team of qualified veterinarians, and standards from sanitary authorities are regularly met.

The founder birds of the captive population were born from eggs collected in the wild. All other birds were produced through a carefully planned breeding program aiming to equalize representation of founders and minimize inbreeding [39]. Controlled breeding was implemented via artificial insemination (see [37] for details). The pedigree used in this study consists of 5692 birds born between 1986 and 2009 (see detailed information on pedigree in Table 1). The breeding adults were housed in individual outdoor cages (cage size: 2 mx4 m) but could see or hear their conspecifics.

**Table 1:** Basic statistics describing the pedigree.

	Ejaculate size	Courtship display rate	Number of eggs	Sperm viability
Number of records	2414	3383	3569	1704
Pedigree depth	8	8	8	7
Founders	170	269	192	117
Number of maternities	2233	3106	3375	1571
Number of paternities	2210	3080	3349	1563
Relatedness of 0.5^a^	4411	6464	6504	2978
Relatedness of 0.25^b^	26492	44181	45203	18606

The pedigree has been pruned in order to retain only individuals informative for the traits under study (function pedantics in R [40], details per age class in S1 Text). Relatedness is calculated among all pairs of individuals, here a pair is any dyad of individuals from the population. More details on pedigree can be found in S1 Text.

^a^parent-offspring or fullsibs

^b^half sibs, nieces or nephews, grandparents / grandchildren

### Courtship Display Rate

During the breeding season, captive males exhibit a complex courtship display combining conspicuous visual and acoustic components similar to those observed in the wild (see [41] for description of courtship display). This energy-demanding behavior of males is monitored for all males three times every day of the year (dawn, early morning and afternoon), and the absence or presence of display was recorded. We defined the courtship display rate as the total number of days each male was observed displaying during a given year. We differ in the definition used in [36] in that the authors of that study focused on sexual display effort among birds that were collected for sperm. Here, we included the information of an absence of display for birds that were alive in a given year and had been known to display in a previous year but also for birds that displayed but did not produce sperm (courtship display rate as defined in [37]). The data on courtship display consist of 8129 observations for 2468 males.

### Ejaculate size and viability of sperm

If a courtship display was observed, the male was stimulated with a dummy female to begin copulation. A Petri dish was positioned under the dummy female to collect the ejaculate. Semen was transferred into an Eppendorf tube and directly brought to an adjacent laboratory to measure ejaculate size and quality. Each ejaculate was diluted in Lake 7.1 diluent, and the number of spermatozoa was assessed using a spectrophotometer analysis [42,43]. A total of 1692 males were sampled repeatedly each year, for a total of 105 538 ejaculates. The number of living sperm and their morphology were assessed using an eosin-nigrosin method (see [44] for details). First, the semen was diluted and eosin-nigrosin stained. Then, the morphology of a minimum of 100 sperm was inspected using a light microscope (X1000). Sperm with double flagella, a swollen membrane or extended nucleus were considered to be aberrant. The data used for sperm quantity were the same as those used in [36]. For sperm viability, Preston et al. [36] focused on the number of non-aberrant sperm, where we were interested in the viability of sperm calculated as the number of non-aberrant and living sperm over the total number of sperm produced (similarly to [37]). The viability parameters were thus calculated as the number of non-aberrant living sperm over the total number of sperm produced. A total of 6210 estimates of sperm viability were assessed for 1144 males.

### Number of eggs produced

Females were inseminated with an average of 12 x 10^6^ spermatozoa. Eggs were collected daily to avoid brooding and placed in incubators for an incubation period of 23 days. The cumulative number of eggs laid per year was used as a proxy of female fecundity. A total of 9575 annual observations were recorded for 3013 females.

### Statistical analysis

The oldest individuals in the dataset were 23 years old; however, because of the scarcity of data for birds older than 15 years and to avoid extrapolating beyond the support of the data, we discarded the observations for those birds between 16 and 23 years of age for all analyses (less than 1% of the amount of data for all traits). These data points from individuals older than 15 years represent less than 10 males that were genetically unrelated and less than 30 females that were weakly genetically related, so no genetic information could be determined from these age classes (S1 Text). The cut-off was kept the same for all analyses. In the wild, the maximum breeding age is assumed to be approximately 10 years [45]. In Morocco, the oldest wild male monitored is 13 years old and the oldest wild female monitored is 11 years old, but their breeding status is unknown (results from GPS monitoring, YH unpublished data).

### Phenotypic senescence

Phenotypic senescence in all traits was investigated using generalized mixed models with a Poisson distribution. Each model included individual identity as a random effect to account for repeated measurements as well as Age (in years) and Age^2^ (in years) as fixed effects to allow for non-linear variation of the traits with age (trait specific fixed effects were also added as described below in animal models).

### Quantitative genetic analysis: General method, data selection and statistical framework

The genetic architecture of aging was explored using “animal models” [46,47]. Animal models partition the phenotypic variance into its different components, namely, additive genetic variance, permanent environment variance estimated by repeated measures of the same individual, and residual variance. The partitioning of inter-individual variance into its additive genetic and other components can be carried out due to the information from multigenerational pedigrees [46,47]. To ensure the accuracy of estimates, we used two methods and report (1) discrete estimates of additive genetic variance within each age class and (2) continuous estimates of additive genetic variance according to age.

Sperm viability data were transformed using the arc-tangent function and analyzed assuming a Gaussian distribution. Ejaculate size, courtship display rate and egg production were Poisson distributed count data. In linear model analyses, the common way to address skewed Poisson distributed data is to log-transform data to obtain a Gaussian distribution. Nevertheless, transformation is not recommended for count data [48]. Although non-Gaussian data can be better addressed using a Bayesian implementation of the animal model [49], a problem is the difficulty of assessing the significance of variance components in a Bayesian framework. This is especially so in the case of random regression (see S3 Text for details) and is the reason we used both a Bayesian and a frequentist framework when performing random regressions (see “Random Regression Animal Model” section). For that reason, animal models were run using both the MCMCglmm package [50] using R for statistical computing [51] and ASReml software [52].

The convergence of the Bayesian models was assessed by (i) graphically checking the posterior estimates and (ii) ensuring that the autocorrelation of all parameter estimates along the MCMC chain was lower than 0.05. Low autocorrelation was achieved using a minimum of 1 200 000 iterations with a burning in of 200 000 and a thinning of 1000, but the number of iterations and thinning were increased in case of autocorrelation. We used parameter expanded priors in all analyses to avoid information from the prior, with one exception for the random regression animal model of courtship display rate, for which we used a prior designed as V = diag(n)*(Vp/3) and nu = n (where n is the number of traits, nu is the degree of freedom and Vp is the phenotypic variance). However, when comparing results from models run with a parameter expanded prior or a slightly informative prior for other traits, the results were similar (results not shown).

### Animal model with discrete age-classes

To estimate age-specific additive genetic variance with a sufficiently large sample size, age-classes were considered. For the courtship display rate and egg production data, the first eight age-class observations were split according to age, whereas in the 9^th^ group, observations were pooled into one group for 9-to-15-year-old individuals (but defining the last age class as 9–23 years did not change the results; results not shown). For ejaculate size and sperm viability, 8-year-old individuals were also pooled into a last age-class because senescence starts early and we needed to ensure sufficient power. This resulted in a sample of at least 100 individuals in each age class (except for sperm viability, S1 Table). The ability to accurately estimate additive genetic variance in older age classes was assessed using a power analysis (see details in S1 Text).

The ideal model would be a multivariate (9x9 **G**-matrix) model estimating variances and covariances between all age-classes [16,17]. Unfortunately, using a Bayesian framework with this model would take an inordinately long time to run, and singularities occurred when this model was run using ASReml software even after reducing the number of age-classes. Thus, we ran multiple univariate and bivariate models instead.

The univariate model used was,

$$y\;=\;\mu \;+\text{ X}\beta +{\text{ Z}}_{1}a\;+{\text{ Z}}_{2}pe\;+\text{ I}e$$ (1)

where **y** was the vector of phenotypic observations of all individuals, μ was the mean phenotype, X was the design matrix relating individuals to fixed effects **β**, Z_1_ was a design matrix relating individuals to **a**, the vector of breeding values, Z_2_ was a design matrix relating individuals to **pe**, the vector of permanent environment effect, I was the identity matrix and **e** was the vector of residual error.

For courtship display rate and egg production, each individual had a single annual record in the first eight age classes; thus, there was no need to account for repeated measures and we did not include a permanent environment term in these analyses. For the oldest age class (9–15), we included a permanent environment effect and age fitted as a fixed effect because measurements were repeated for individuals.

For ejaculate size and sperm viability, several measures were available for the same individual each year, so we fitted an identity random effect to account for these repeated measurements. To account for the potential impact of the frequency of collection, we used the number of days elapsed since the last ejaculate as a fixed effect. Moreover, seasonal variation in ejaculate size was taken into account with the number of days since the first ejaculate of the year fitted as a fixed effect. Note thatconsidering quadratic and cubic effects did not change the results. As year of collection and age were confounded with higher ages in our data set, year of collection was not fitted in our animal models.

For those models using a Poisson distribution, DIC cannot be used to assess the best model (see S3 Text for details) and, because a variance component is constrained to be greater than 0, the credible intervals cannot be used to formally assess the significance of Va. Nevertheless, two lines of evidence allowed inference of the significance of additive genetic variance. First, we used parameter-expanded priors, so no information was gained from the prior to estimate Va. Second, we inspected the posterior distribution and concluded that Va was greater than 0 when the credible interval was far from 0 and that Va was extremely low when the credible interval collapsed to 0. We characterized the variation in additive genetic variance across ages using pair-wise post hoc-tests on posterior distributions (methods detailed in S2 Text).

Because Va was estimated from models that assumed a Poisson distribution, the estimate of heritability should account for the link between mean and variance. We calculated Poisson heritability (hereafter called Poisson-heritability) as

$$\;\;\;\;\;\;\;\;\;\;\;h^{2}=\frac{V_{a}}{V_{a}+V_{pe}+V_{r}+\;log\left\{\frac{1}{exp\left(Xage\right)}+1\right\}},$$ (2)

where (Xage) was the raw mean of specific age-classes (see [53,54] for more details).

To test for the existence of within trait antagonistic pleiotropy, we estimated the matrix of additive genetic variance-covariance (**G**-matrix) of a trait among age classes. This matrix was estimated by running bivariate animal models on discrete age-classes as used in univariate models. In bivariate models, the fixed effects remained the same as in the corresponding univariate models, but the vectors of random effects were replaced by the matrices of variance-covariance (see S2 Text for further details).

### Random regression animal models (RRAM)

Random regressions were used to model the phenotypic variance and covariance as a function of a continuous variable (in this case, age) [46]. The use of existing family relationships between individuals in the form of a pedigree allowed us to separate the phenotypic variance into additive genetic variance and non-genetic variance. Using the random regression animal model (RRAM), we partitioned individual variation across age groups as a function of additive genetic variance and environmental effects. In comparison to univariate animal models, the RRAMs model accounts for the individual covariance patterns among age-specific traits.

To select the best RRAM model, we followed a two-step selection strategy as in Brommer et al. [26] using log transformed data (except for sperm viability). We first assessed the model that best described individual variation across ages and then proceeded to assess patterns of additive genetic variance components with age. Model selection was performed using a likelihood ratio test (LRT), where twice the difference in the log-likelihood of models was compared to a Χ^2^ distribution with degrees of freedom equaling the difference in the number of parameters between the two models.

A random regression model was used, i.e.,

$$y\;=\;\mu \;+\text{ X}\beta \;+\text{ f}\left(id,\text{ i},\;age_{ST}\right)\;+\;e$$ (3)

where **y** is the vector of phenotypic observations on all individuals, **μ** is the mean phenotype, X is the design matrix relating individuals to fixed effects **β** and **id** is the individual effect. For all traits, age was fitted as a factorial fixed effect to control for differences in phenotypic values across ages. For ejaculate size, the frequency of collection and seasonal variation defined above were also fitted as fixed effects. Legendre polynomial functions (f) were used to model individual variance, f(**id**, *i*, **age**_**ST**_), where i was the order of Legendre polynomial and age_**ST**_ was the age parameter scaled between -1 and 1 (1 year old and 15 years old, respectively) to meet the Legendre polynomial conditions of application; **e** is the residual variance covariance matrix. We allowed for heterogeneity in residual variance by fitting age-specific residual variances (i.e., 15 residual units), and the residual correlations between age classes were constrained to zero.

We started by comparing the fit of a model assuming first-order polynomial variation between individuals (i = 1 in Eq 3) to the fit of a model assuming a constant between individual variation across ages (i = 0 in Eq 3). Then, we sequentially increased the polynomial order of the model until the increase in polynomial order was not supported and defined i_best_, the order of Legendre polynomial that best described individual variation across ages.

From this best model, we partitioned the individual variance into a function of additive genetic variance and permanent environment variance as,

$$y\;=\;\mu \;+\text{ X}\beta \;+\text{ f}\left(a,\text{ j},\;age_{ST}\right)\;+\text{ f}\left(pe,{\text{ i}}_{\text{best}},\;age_{ST}\right)\;+\;e$$ (4)

where the permanent environmental (pe) variance was of the same polynomial order as the previous individual effect (**id**), and the polynomial order for **a**, breeding values, was sequentially increased starting from 0. Finally, the best animal model was also run with a Poisson distribution using the MCMCglmm package.

For each trait, the age-specific variance-covariance matrix of additive genetic variance (**G**) was obtained from back-transformation of the Legendre polynomial function *f(* ***a***, *j*, **age**_**ST**_ *)*. The variance estimates are provided on the liability scale (see details of the back-transformation in S3 Text).

## Results

### Phenotypic senescence

**Fig 1:**
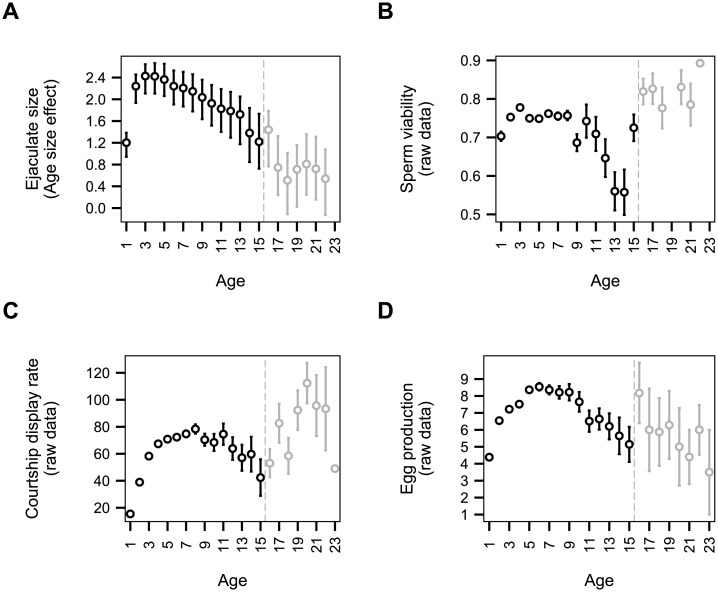
Age-related variation of houbara bustard reproductive traits. As ejaculate size (A) measures were intra-annually repeated raw data cannot be used to assess age-related variation. Age-related size effect was estimates using mixed model on phenotypical data (see Material and method and Results; posterior mode ± Credible Interval). For Courtship display rate (B), Egg production (C) and Sperm viability (D), raw data were plotted (mean ± SE). All traits showed phenotypic senescence (see Results). The ages in grey were not included into analyses.

All traits showed patterns of senescence at the phenotypic level (Fig 1). Note that we discarded observations from those birds between 16 and 23 year of age prior to performing the analyses of phenotypic senescence. In line with results from [36], sperm viability and ejaculate size decreased with age (Fig 1A, Table 2) from ages 8 and 5, respectively. For courtship display rate, a slight decline was observed by Preston et al. [36] for individuals between 12 and 15 years of age, preceding an increase in effort for birds older than 16 years. However, biological significance of this terminal increase is subject to caution due to the very low sample sizes (3 to 7 individuals) of the 16+ year old age class which was not used in the analyses. Thus, there was moderate senescence in courtship display rate (Fig 1, Table 2) from age 9. Finally, the number of eggs produced by females strongly declined at the phenotypic level when females reached 9 years of age (Fig 1, Table 2).

**Table 2:** Phenotypic senescence for breeding traits of the houbara bustard as evaluated through Age and Age² effects. The date since the last ejaculation (Dsle) and the date of collect (Doc) were only fitted for ejaculate size. The values correspond to the posterior mode ± Credible Interval.

	Ejaculate size (x10^-2^)	Sperm viability (x10^-2^)	Courtship display rate (x10^-1^)	Egg production (x10^-2^)
Age	23.37 [22.29:24.44]	40.37 [39.72:41.18]	12.85 [12.55:13.15]	56.84 [55.35:58.15]
Age^2^	-2.06 [-2.17:-1.97]	-3.08 [-3.18:-2.96]	-1.03 [-1.05:-0.99]	-3.70 [-3.85:-3.55]
Dsle	1.67 [1.52:1.83]			
Doc	0.16 [0.14:0.17]			
Individual variance	101.12 [93.32:109.02]	1.88 [0.96:2.81]	10.37 [9.19:11.54]	50.51 [46.79:55.12]
Residual variance	52.68 [52.12:53.25]	9.11 [8.37:10.10]	11.33 [10.80:11.86]	34.17 [32.08:36.01]

### Patterns of additive genetic variance

Except for sperm viability, additive genetic variance (Va) was found in all traits across the entire data set and within age classes (S1 Table). For sperm viability, the random regression assuming an interaction between individual variance and age did not converge.

**Fig 2:**
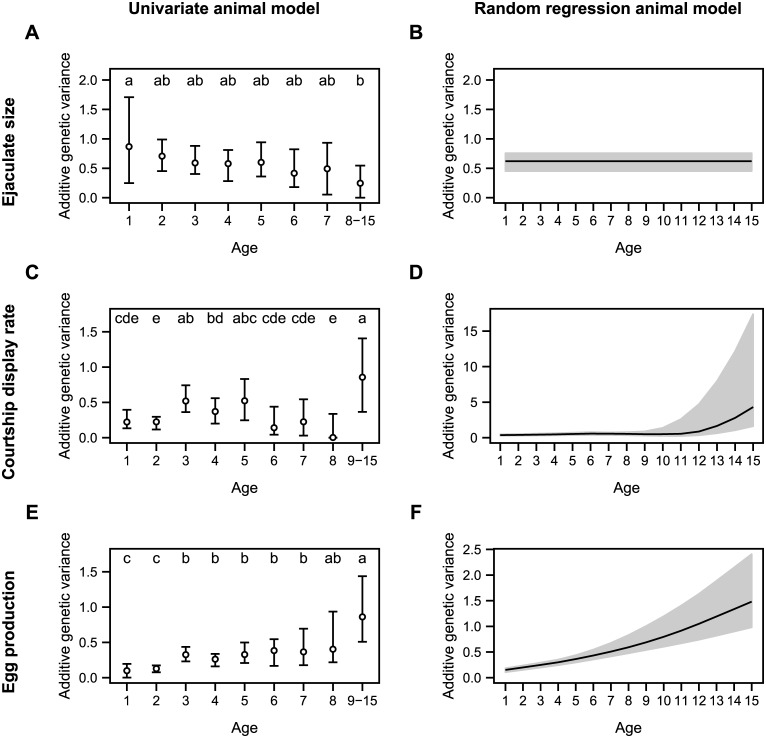
Variation of additive genetic variance across ages. Additive genetic variance was estimated using univariate animal models (left column) and random regression animal models (right column) for (A-B) ejaculate size, (C-D) courtship display rate and (E-F) egg production. For univariate animal models, circles represent the posterior mode estimation of additive genetic variance (with their 95% credible interval). Different letters represent significant differences between posterior estimates (see S2 Text for post-test details). For random regression animal models, the posterior mode estimates and their 95% credible interval are represented by black lines and associated grey area. All variance estimates are given on the latent scale and not back-transformed to the phenotypic scale. Variance estimates and fixed parameter estimates are provided in S1 and S2 Tables. Models were run using MCMCglmm package [50].

**Fig 3:**
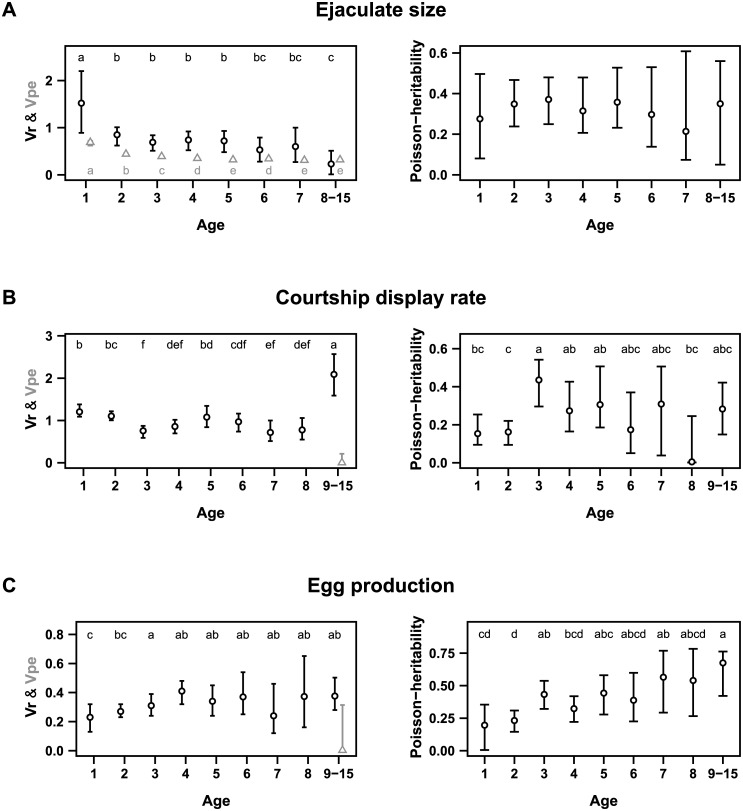
Variation of non-genetic variance components across ages. Vr (residual variances), Vpe (permanent environment variance) and Poisson-heritability are provided for (A) ejaculate size, (B) courtship display rate and (C) egg production. In the left column, Vr are presented with black circles and Vpe with grey triangles. The letters represent significant differences between posterior estimates (see S2 Text for post-test details). Models were run using MCMCglmm package [50].

For ejaculate size, the posterior estimates of additive genetic variance were greater than 0 in all age classes and Va was lower for older birds (Fig 2A, S1 Table). In contrast to our hypotheses, we found a slight linear decrease of Va across ages when performing the pair-wise tests among univariate posterior estimates (Fig 2A). Model selection for random regression animal models showed the best support for a constant Va across ages (Fig 2B, Table 3, S3 Text). The permanent environmental variances, residual variances and Poisson-heritabilities were also constant with age (Fig 3A).

**Table 3:** Results of the selection strategy of the best random regression animal model for ejaculate size. LogL, the Log Likelihood of the model, LRT the likelihood ratio test, d.f., the degree of freedom defined as the number of random term(s) (variance(s) and covariance(s)) added for fitting each model in comparison of previous model. Note that f(id, 3, age_ST_) and f(a, 2, age_ST_) + f(pe, 2, age_ST_) did not converge. A plot of the best model can be found in Fig 2 (using MCMCglmm estimates) and in S3 Text (using ASReml estimates).

Models no.	Random regression model	LogL	LRT	d.f.	P-value
1	f(id, 0, age_ST_)	-29124.09			
2	f(id, 1, age_ST_)	-24630.96	8986.268	2	<0.001
3	f(id, 2, age_ST_)	-23200.67	2860.58	3	<0.001
**4**	**f(a, 0, age**_**ST**_ **) + f(pe, 2, age**_**ST**_ **)**	**-23114.55**	**172.24**	**1**	**<0.001**
5	f(a, 1, age_ST_) + f(pe, 2, age_ST_)	-23112.94	3.22	2	0.20

For courtship display rate, the posterior estimates of additive genetic variance were clearly greater than 0 for all age classes except for the 8-year-old age class (S1 Table). The pair-wise test between univariate posterior estimates showed significant variation in Va among age classes. Although Va estimates were low for precocious birds (1 and 2 years old) and mature individuals (6, 7 and 8 years old), they were high for young individuals (3, 4 and 5 years old) and the oldest bird age-class (9–15) (Fig 2C, S1 Table). In the context of genetic theories of aging, there was a significant increase in additive genetic variance in the oldest age class. The random regression models supported this result because they provided the best support for a second-order polynomial variation of Va (Table 4) with a late increase in additive genetic variance (Fig 2D, S3 Text) from 10 years old onward. Permanent environment and residual variances increased in the older age class, whereas the Poisson-heritability remained constant across ages (Fig 3B).

**Table 4:** Results of the selection strategy of the best random regression animal model for courtship display rate. LogL, the Log Likelihood of the model, LRT the likelihood ratio test, d.f., the degree of freedom defined as the number of random term(s) (variance(s) and covariance(s)) added for fitting each model in comparison of previous model. Note that f(indiv, 3, age_ST_) did not converge A plot of the best model can be found in Fig 2 (using MCMCglmm estimates) and in S3 Text (using ASReml estimates).

Models no.	Random regression model	LogL	LRT	d.f.	P-value
1	f(id, 0, age_ST_)	-5653.74			
2	f(id, 1, age_ST_)	-4979.09	255.04	2	<0.001
3	f(id, 2, age_ST_)	-4889.55	179.08	3	<0.001
4	f(a, 0, age_ST_) + f(pe, 2, age_ST_)	-4819.06	140.98	1	<0.001
5	f(a, 1, age_ST_) + f(pe, 2, age_ST_)	-4812.58	12.96	2	0.002
**6**	**f(a, 2, age**_**ST**_ **) + f(pe, 2, age**_**ST**_ **)**	**-4803.96**	**17.24**	**3**	**<0.001**

For the number of eggs produced, additive genetic variance increased significantly across ages (S1 Table). This increase was supported by the pair-wise test between univariate posterior estimates (Fig 2E). For egg production, the random regression models provided the best support for first-order polynomial variation of Va with a monotonic increase of Va with age (Fig 2F; Table 5; S3 Text). The residual variances remained constant and the Poisson-heritability increased across ages (Fig 3C).

**Table 5:** Results of the selection strategy of the best random regression animal model for egg production. LogL, the Log Likelihood of the model, LRT the likelihood ratio test, d.f., the degree of freedom defined as the number of random term(s) (variance(s) and covariance(s)) added for fitting each model in comparison of previous model. Note that f(indiv, 2, age_ST_) did not converge. A plot of the best model can be found in Fig 2 (using MCMCglmm estimates) and in S3 Text (using ASReml estimates).

Models no.	Random regression model	LogL	LRT	d.f.	P-value
1	f(indiv, 0, age_ST_)	-2719.19			
2	f(indiv, 1, age_ST_)	-2598.53	241.32	2	<0.001
3	f(a, 0, age_ST_) + f(pe, 1, age_ST_)	-2446.88	303.30	1	<0.001
**4**	**f(a, 1, age**_**ST**_ **) + f(pe, 1, age**_**ST**_ **)**	**-2412.2**	**69.36**	**2**	**<0.001**

### Between-age genetic correlations

Bivariate animal models provided no evidence for negative genetic correlations between the first age class (1 year old) and the oldest age class in any of the traits. For ejaculate size and courtship display rates, genetic correlations between young birds (1 year old) and the oldest birds were low and not significantly different from zero. For the number of eggs produced, these genetic correlations remained significantly positive between the young and old birds (Tables 6–8).

**Table 6:** Results of genetic correlations between age-classes for ejaculate size.

	Age 1	Age 2	Age 3	Age 4	Age 5	Age 6	Age 7
Age 2	0.82 [0.57:1.00]						
Age 3	0.88 [0.67:0.98]	0.95 [0.86:0.99]					
Age 4	0.85 [0.53:0.95]	0.95 [0.86:0.99]	0.95 [0.83:0.99]				
Age 5	0.72 [0.36:0.91]	0.93 [0.81:0.99]	0.89 [0.77:0.99]	0.97 [0.87:1.00]			
Age 6	0.71 [0.16:0.94]	0.88 [0.72:0.97]	0.95 [0.85:1.00]	0.88 [0.73:0.98]	0.94 [0.77:0.99]		
Age 7	**0.57 [-0.11:0.98]**	0.91 [0.65:0.99]	0.95 [0.77:1.00]	0.98 [0.84:1.00]	0.97 [0.82:1.00]	0.99 [0.91:1.00]	
Age 8–15	**0.19 [-0.51:0.74]**	0.79 [0.41:0.97]	0.83 [0.61:0.99]	0.79 [0.55:0.97]	0.84 [0.63:1.00]	0.94 [0.73:1.00]	0.96 [0.74:1.00]

Genetic correlations in bold were not significantly different from 0.

**Table 7:** Results of genetic correlations between age-classes for courtship display rate.

	Age 1	Age 2	Age 3	Age 4	Age 5	Age 6	Age 7	Age 8
Age 2	0.81 [0.57:0.96]							
Age 3	0.45 [0.14:0.69]	0.89 [0.73:0.98]						
Age 4	**0.29 [-0.04:0.71]**	0.84 [0.56:0.96]	0.92 [0.81:0.97]					
Age 5	**0.32 [-0.08:0.71]**	0.75 [0.48:0.92]	0.74 [0.58:0.89]	0.89 [0.70:0.96]				
Age 6	**0.22 [-0.25:0.68]**	0.70 [0.27:0.92]	0.68 [0.37:0.92]	0.81 [0.60:0.99]	0.80 [0.52:0.96]			
Age 7	**0.20 [-0.18:0.61]**	0.56 [0.08:0.74]	**0.24 [-0.22:0.49]**	0.54 [0.24:0.82]	0.62 [0.22:0.82]	0.64 [0.20:0.79]		
Age 8	**0.22[-0.37:0.96]**	**0.5[-0.41:0.92]**	**0.66[-0.24:0.94]**	0.77[0.19:0.99]	**0.91[-0.02:1]**	**0.66[-0.29:0.98]**	**0.74[-0.35:0.99]**	
Age 9–15	**-0.22[-0.64:0.5]**	0.59[0.04:1.00]	**-0.48[-0.89:0.77]**	**0.2[-0.51:0.99]**	**0.62[-0.27:0.99]**	0.85[0.32:0.99]	0.92[0.48:0.99]	0.92[0.16:0.99]

Genetic correlations in bold were not significantly different from 0.

**Table 8:** Results of genetic correlations between age-classes for the number of eggs produced.

	Age 1	Age 2	Age 3	Age 4	Age 5	Age 6	Age 7	Age 8
Age 2	0.93 [0.66:1.00]							
Age 3	0.90 [0.71:1.00]	0.91 [0.77:0.98]						
Age 4	0.90 [0.71:1.00]	0.91 [0.79:0.99]	0.90 [0.82:0.97]					
Age 5	0.92 [0.70:1.00]	0.83 [0.66:0.96]	0.97 [0.88:1.00]	0.94 [0.85:0.99]				
Age 6	0.84 [0.48:0.98]	0.67 [0.30:0.88]	0.93 [0.75:0.98]	0.96 [0.85:1.00]	0.95 [0.84:1.00]			
Age 7	0.91 [0.42:0.99]	0.53 [0.19:0.91]	0.64 [0.44:0.95]	0.91 [0.67:0.99]	0.94 [0.80:0.99]	0.98 [0.89:1.00]		
Age 8	**0.51[-0.23:0.93]**	**0.25[-0.08:0.82]**	0.84[0.47:0.98]	0.74[0.37:0.94]	0.80[0.57:1.00]	0.92[0.66:1.00]	0.96[0.77:1.00]	
Age 9–15	**-0.01[-0.74:0.92]**	**0.67[-0.21:0.98]**	**0.43[-0.01:0.94]**	0.84[0.46:0.98]	0.62[0.22:0.95]	0.71[0.4:0.95]	0.98[0.68:1.00]	0.89[0.62:0.99]

Genetic correlations in bold were not significantly different from 0.

When genetic correlations between young birds and birds of other age classes were excluded, genetic correlations varied between 0.24–0.92 for courtship display rate and 0.52–0.98 for egg production. For both traits, genetic correlations decreased as age differences increased. For ejaculate size, genetic correlations ranged from 0.79–0.99, and the decrease of these correlations as age differences increased seemed moderate in comparison with those of other traits (Tables 6–8).

Because of the extremely low additive genetic variance of sperm viability across age classes, the genetic correlations between age-classes were not calculated for this trait (Tables 6–8).

## Discussion

All reproductive traits of the houbara bustard investigated here showed senescence at the phenotypic level corroborating previous findings on this species [36]. These senescence patterns are in agreement with results obtained in a wide range of taxa on behavioral sexual traits (e.g., rut of red deer: [17]; mating rate of antler flies *Protopiophila litigata* [55]), sperm traits (e.g., *Homo sapiens*: [56]; *Hirundo rustica*: [57]; *Gallus gallus domesticus*: [58]) and egg production traits (*Gallus gallus domesticus*: [59]); see [60] for review. Surprisingly, quantitative genetic analyses of reproductive traits of the houbara bustard showed very different patterns among traits. The expected increase in additive genetic variance was found only for courtship display rate and egg production, but not for sperm traits (ejaculate size, sperm viability). Furthermore, positive genetic correlations between age classes revealed that the animal models did not detect antagonistic pleiotropy for these traits.

### Increased Va with age: the quantitative genetics of aging in courtship display rates and egg production

Both courtship display rate and egg production showed a terminal increase in additive genetic variance, consistent with genetic theories of senescence [8,9]. Although the genetic variance increased monotonically with age for the annual number of eggs laid, this increase was non-monotonic for courtship display rate. The result for egg production was consistent with theories of senescence predicting an increase in additive genetic variance from the age of primiparity, ca. four years old in the houbara bustard.

In turn, the pattern of additive genetic variance was more complex for courtship display rate. Although high additive genetic variance in young and older age classes and low genetic variance in mature individuals is apparent is some species [15,16], additive genetic variance for courtship display rate was also low in very young houbara bustards. Sequential recruitment in the breeding population and experience may contribute to this pattern. In the early part of life (1–2 yr old), some males start displaying at a much lower rate than older males (Fig 1), leading to similar levels of display among unrelated birds and resulting in relatively low Va. Accordingly, the residual variance is higher than Va in young birds and suggests an important non-genetic process explaining the differences in courtship display rate among young birds. At 3 years old, a large majority of birds become sexually mature, Va is high and so is the Poisson-heritability. Decreasing Va and Poisson-heritability after 5 years of age (mature birds) may be explained by the increasing importance of male experience, and diminishing the relative importance of the genotype on courtship display rates. In line with this, a large number of studies have shown that age-specific breeding performance improves with age [61,62]. Finally, in terms of aging patterns, the later than expected decrease in performance is now a classic result [28,63] along with a late increase in Va [15,64]. The observed increase in Va at old ages is consistent with genetic theories of senescence because it corresponds to the age-class where a decrease in performance was found.

For both courtship display rates and number of eggs produced, positive genetic correlations among early and late expression of the traits do not support within trait antagonistic pleiotropy. One reason could be that in the animal model, the effects of all loci are averaged together so that if AP occurs among some loci, it may not be detected [65]. Hence, further studies are required, notably to investigate the age-related variation of genetic correlations between reproductive traits and perhaps at the genome level to fully assess the generality of antagonistic pleiotropy.

### Sperm traits: the absence of late terminal increase in additive genetic variance

In contrast with courtship display rates and number of eggs, no increase in additive genetic variance was detected for sperm traits. Altogether, the stability of genetic variance in ejaculate size and the positive genetic correlations between the early and late expressions did not support either the MA or AP theories [8].

For sperm viability, the amount of additive genetic variance was extremely low in the houbara bustard (S1 Table, [37]). A low level of genetic variance for the same trait was found in cockroaches (*Nauphoeta cinerea*) [66]. However, as a significant heritability was also detected for sperm viability in other species, the data are still too scarce to assess the generality of this finding. A recent review in birds, mammals and insects [67] showed a lower overall heritability of sperm production traits (e.g., ejaculate size) than sperm morphology traits (e.g., sperm viability). Although a genetic variance increasing from zero in young adult individuals to non-zero in old ages has already been found [15], we did not find any evidence of such a terminal increase in sperm viability. Sperm viability has been suggested to be a better predictor of paternity than the total number of sperm (see [68] for review), so strong selection pressures on sperm viability can explain the very low additive genetic variance in young and middle ages [6]. The persistence of low additive genetic variance in old age implies that genetic theories of aging cannot alone be invoked to explain aging in this trait.

Three methodological issues may explain the lack of age-related genetic patterns of both sperm traits, i.e. ejaculate size and viability. First, by reducing the genotype differences among older birds, selective disappearance can reduce genetic variance at old ages compared to that at younger ages. To assess the importance of selective disappearance in shaping these patterns, all univariate animal models were re-run after the removal of the animals that had died during the time period covered by our study. A few birds died during the period of the study, so it was not possible to run a model with longevity as a covariate because the data were right-censored. The removal of dead animals from the analyses allowed us to test whether an increase in Va was masked by a negative correlation between the longevity of individuals and their poor reproductive performance. The results provided in S1 Fig showed that age-related variation in Va was unchanged when dead animals were removed from the analyses. Second, the absence of a terminal increase in additive genetic variance in old ages may reflect the reduction of statistical power due to the reduction of sample size. The scarcity of data in old ages is a common issue in senescence studies that can be problematic [69]. To ensure that the lack of increase was not due to the scarcity of data, we ran a power analysis and found that Va may be underestimated for sperm viability but not for ejaculate size (S1 Text). Third, as the decrease in the mean value of a trait is mathematically linked to a decrease in its variance, a decrease of trait value with age might be responsible for a low Va. To test for this potential artifact on ejaculate size and sperm viability, for each age class, we divided the trait values by their mean (evolvability [70]) and re-ran univariate models, but no increase in Va was detected (Figure A in S2 Fig).

How can the existence of senescence be explained in the absence of genetic changes over the lifespan? Evolutionary biologists interested in the evolution of the mortality senescence of *Drosophila* have found results that challenge predictions of both MA and AP genetic theories of senescence (e.g., [20–25]). In natural populations, two studies of collared flycatchers (*Ficedula albicollis*) [27] and common gulls (*Larus canus*) [26] also found a pattern of senescence on fitness associated with a constant Va with age.

The first explanation relies on the assumptions that were used by theoretical models to predict age-related patterns of genetic variance components of MA and AP [8,9]. Although these traditional theoretical models predicted an increase in Va with age, Snoke & Promislow [24] showed that modifying the scale of the mutational effects used in theoretical models where mutations acted multiplicatively instead of additively can influence the expected pattern of variance under MA and even predict a decrease in Va across ages. In the same vein, Rose & Charlesworth [20] postulated that if late-acting mutations have much smaller proportional effect on fecundity than the early acting ones, stability in Va may be detected. From a general view, Moorad & Promislow [71] highlighted that predictions of variance components under the evolutionary theory of senescence largely depend on the starting assumptions of theoretical models.

An alternative evolutionary theory of aging, the reliability theory of aging [25,72,73], is based on the accumulation of damage throughout life and allows for phenotypic senescence with stable genetic variance at old ages. According to this theory, the reliability of a system depends on the reliability and amount of redundancy of these components: high redundancy allows tolerance of the failure of some components. The age–related progressive accumulation of random damage in a redundant system may produce a senescence pattern that does not necessarily require an age-related increase or even the presence of additive genetic variance. As we found for sperm traits, the extreme accumulation of damage can lead to resemblance among individuals at extreme old ages, leading to low phenotypic variance at these ages. Recently, Laird & Sherratt [73] have demonstrated with a theoretical model that senescence may evolve in initially non-senescing ancestral populations by relying only on the reliability theory of aging. This evolutionary theory of aging, taking into account gradual damage, is not exclusive and may coexist with MA and AP as an evolutionary explanation of aging.

### The multi-factorial process of aging

We show contrasting age-related genetic variance patterns among reproductive traits. Although the increase in additive genetic variance for courtship display rates and number of eggs produced fit the mutation accumulation and antagonistic pleiotropy theories of aging, no such genetic signal was observed in sperm performance despite marked phenotypic senescence. To our knowledge, this study provides the first quantitative genetic assessment of aging for sperm performance traits.

The decrease in the force of selection should apply to all traits related to fitness, although the magnitudes of these effects depend on how each particular trait actually covaries with fitness. Together with the difficulty of detecting small ranges of variation using quantitative genetics models, it may explain why we did not detect an increase in the additive genetic variance of sperm traits. Rose et al. [1] argued that the physiology of aging is “moulded and constrained according to the dictates of natural selection shaping adaptation”. In agreement with this concept, the reliability theory of aging may appear to be an interesting theory to explain the results we found in sperm performance traits at the phenotypic and genetic levels.

This diversity of mechanisms might imply that at the population or species level, the senescence of some traits is directly related to additive genetic variance, which may or may not involve trade-offs between ages. Consequently, investigating aging phenotypes as a whole using multivariate analyses rather than a single trait is thus important in shaping an accurate picture of the mechanisms of aging (Chantepie et al. in prep.).

## Supporting Information

S1 Fig Additive genetic variance for (A) ejaculate size, (B) courtship display rate and (C) egg production traits when accounting for selective disappearance. To account for selective disappearance, all birds which died during the time of study were removed from the analyses. We used univariate animal models described in material and method.
(DOCX)

S2 Fig Additive genetic variance for (A) ejaculate size, (B) courtship display rate and (C) egg production traits when the trait values were divided by their mean within each age class. Data were log(x+1) transformed and Gaussian link functions were used into the models.
(DOCX)

S1 Table Results from univariate animal models for ejaculates size trait, courtship display rate, number of egg traits and sperm viability. (DOCX)

S2 Table Results from best random regression animal models for ejaculates size trait, courtship display rate, number of egg traits and sperm viability estimated using MCMCglmm. (DOCX)

S1 Text The number of individuals per age for the traits under study, information on relatedness and power analyses on univariate age-classes. (DOCX)

S2 Text Model parameterisations for bivariate animal models and post-test methodology used for univariate animal models. (DOCX)

S3 Text Details about the random regression animal models. The deviance information criterion (DIC), back-transformation of the Legendre polynomial function into Va and age-related variation of the additive genetic variance plotted from the best random regression animal model found in Tables 3–5 using ASReml software.
(DOCX)
