# Supplementary figures and images for: Quantitative Genetics of the Aging of Reproductive Traits in the Houbara Bustard
Source: PLoS One. 2015 Jul 28;10(7):e0133140. doi: 10.1371/journal.pone.0133140 (PMC4517785; doi:10.1371/journal.pone.0133140)

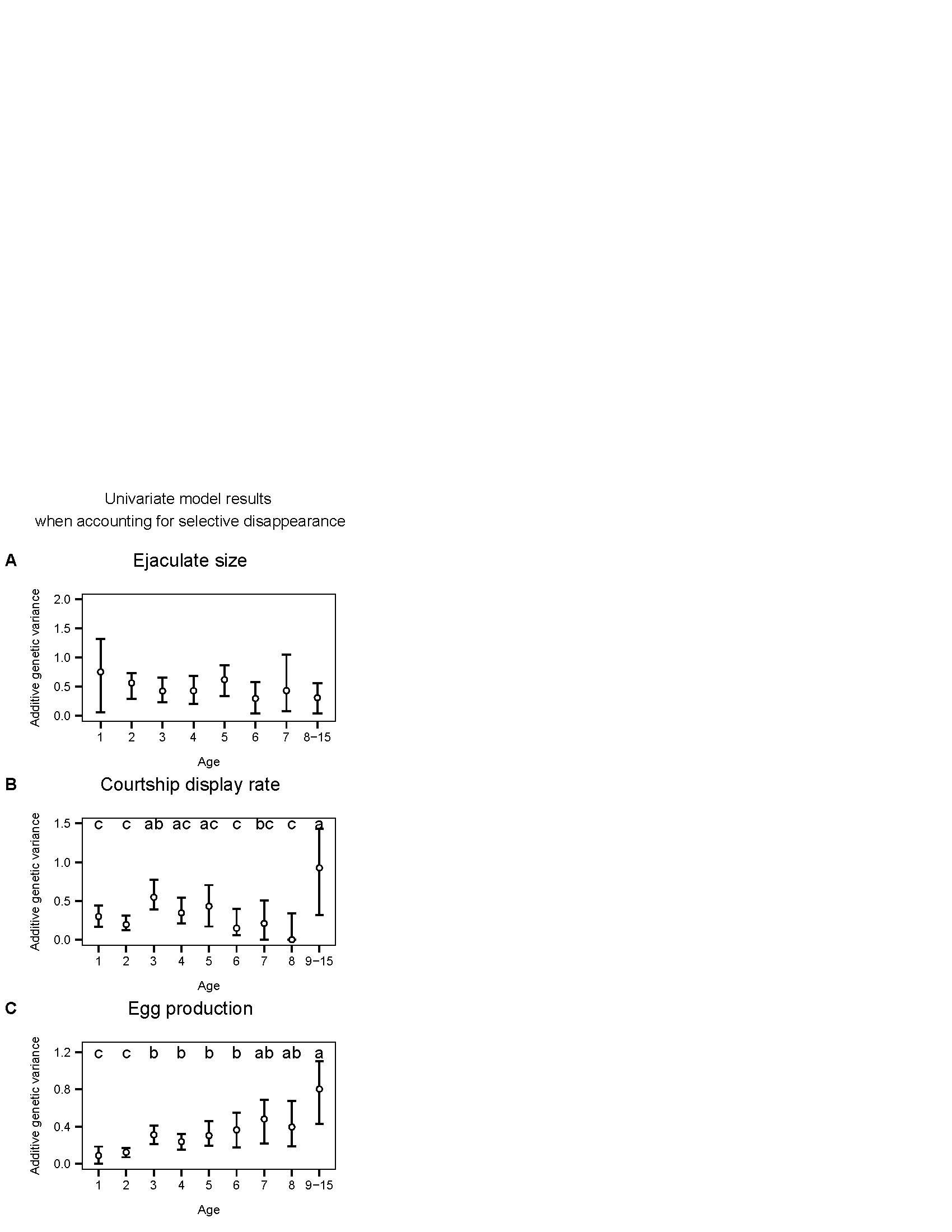

Supplement: S1 Fig — To account for selective disappearance, all birds which died during the time of study were removed from the analyses. We used univariate animal models described in material and method. (DOCX) [file pone.0133140.s001.docx]

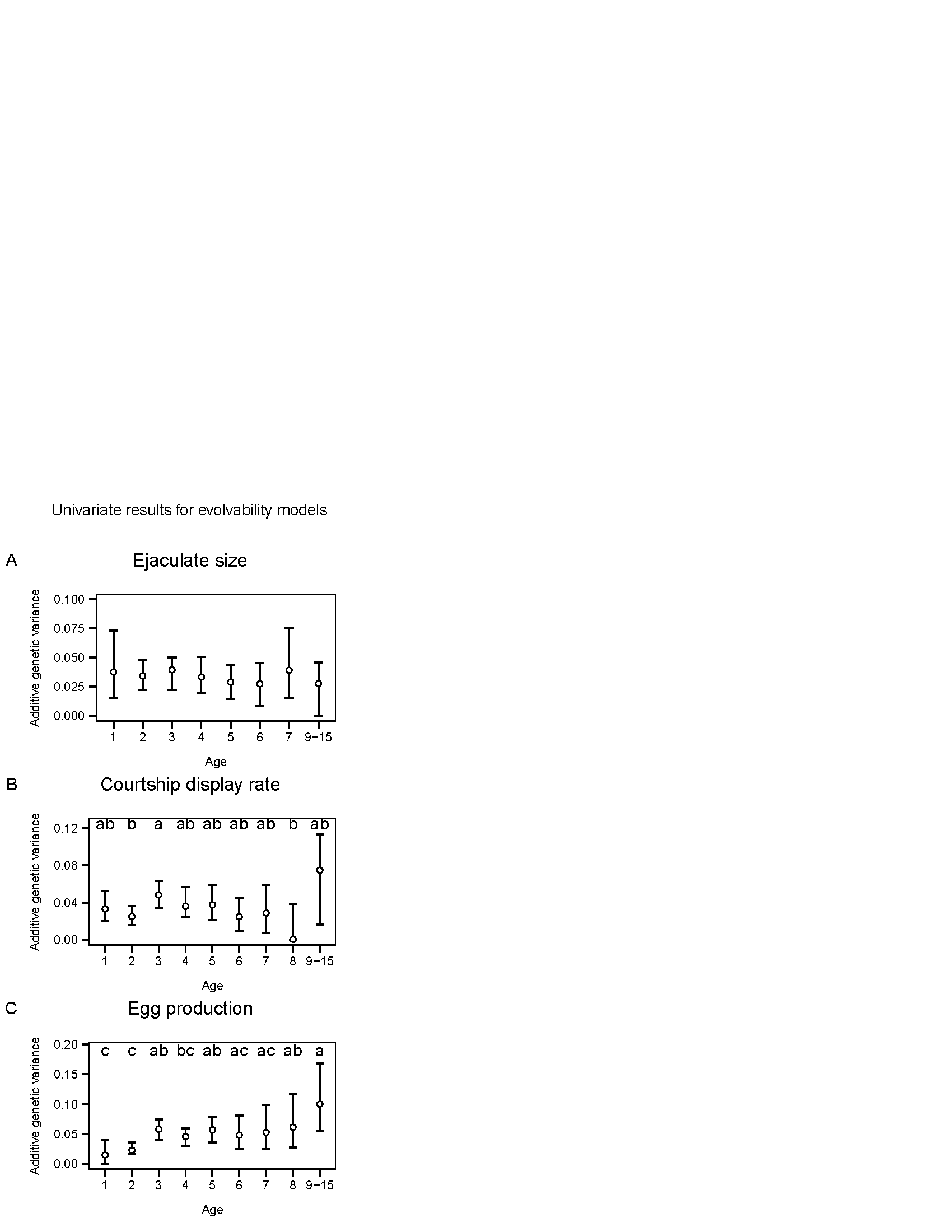

Supplement: S2 Fig — Data were log(x+1) transformed and Gaussian link functions were used into the models. (DOCX) [file pone.0133140.s002.docx]
