# Supplementary material for: Quantitative Genetics of the Aging of Reproductive Traits in the Houbara Bustard
Source: PLoS One. 2015 Jul 28;10(7):e0133140. doi: 10.1371/journal.pone.0133140 (PMC4517785; doi:10.1371/journal.pone.0133140)
Supplement: S1 Table — (DOCX) [file pone.0133140.s003.docx]

Table S1A: Variances component estimates from univariate animal models for ejaculate size.

| **Traits** | **Age** | **Va [95%CI]** | **Vpe [95%CI]** | **Vr [95%CI]** | **Vp [95%CI]** | **h^2^** | **N**  **ind** |
| --- | --- | --- | --- | --- | --- | --- | --- |
| Ejaculate size | 1 | 0.87 [0.25:1.71] | 1.52 [0.89:2.20] | 0.69 [0.62:0.72] | 3.02 [2.73:3.70] | 0.28 [0.08:0.50] | 486 |
|  | 2 | 0.71 [0.45:0.99] | 0.85 [0.62:1.01] | 0.44 [0.43:0.45] | 1.97 [1.79:2.13] | 0.35 [0.24:0.47] | 1175 |
|  | 3 | 0.60 [0.04:0.88] | 0.69 [0.51:0.84] | 0.39 [0.38:0.40] | 1.69 [1.53:1.84] | 0.37 [0.25:0.48] | 1004 |
|  | 4 | 0.58 [0.28:0.81] | 0.74 [0.52:0.92] | 0.35 [0.34:0.36] | 1.60 [1.48:1.82] | 0.31 [0.21:0.48] | 766 |
|  | 5 | 0.60 [0.36:0.94] | 0.72 [0.48:0.93] | 0.32 [0.31:0.33] | 1.62 [1.44:1.81] | 0.36 [0.23:0.53] | 691 |
|  | 6 | 0.42 [0.18:0.82] | 0.53 [0.28:0.79] | 0.34 [0.33:0.34] | 1.35 [1.16:1.57] | 0.30 [0.14:0.53] | 431 |
|  | 7 | 0.49 [0.05:0.93] | 0.60 [0.27:1.00] | 0.31 [0.29:0.32] | 1.33 [1.18:1.70] | 0.21 [0.07:0.61] | 279 |
|  | 8-15 | 0.25 [2.02e-7:0.55] | 0.23 [0.01:0.51] | 0.32 [0.31:0.33] | 0.89 [0.76:1.08] | 0.35 [0.05:0.56] | 166 |
|  | all | 0.68 [0.51:0.85] | 0.42 [0.30:0.51] | 0.52 [0.51:0.52] | 1.60 [1.51:1.72] | 0.42 [0.34:0.50] | 1692 |
| Sexual display effort | 1 | 0.22 [0.13:0.40] |  | 1.20 [1.09:1.38] | 1.47 [1.37:1.62] | 0.15 [0.09:0.25] | 1638 |
|  | 2 | 0.22 [0.12:0.30] |  | 1.10 [1.00:1.22] | 1.28 [1.21:1.41] | 0.16 [0.09:0.22] | 2035 |
|  | 3 | 0.52 [0.36:0.74] |  | 0.76 [0.59:0.87] | 1.25 [1.15:1.39] | 0.44 [0.30:0.54] | 1450 |
|  | 4 | 0.37 [0.20:0.56] |  | 0.86 [0.69:1.02] | 1.22 [1.11:1.36] | 0.27 [0.16:0.43] | 1080 |
|  | 5 | 0.52 [0.25:0.83] |  | 1.08 [0.84:1.34] | 1.57 [1.42:1.83] | 0.31 [0.19:0.51] | 887 |
|  | 6 | 0.14 [0.04:0.44] |  | 0.97 [0.74:1.16] | 1.20 [0.99:1.33] | 0.17 [0.05:0.37] | 552 |
|  | 7 | 0.22 [0.03:0.54] |  | 0.72 [0.51:1.00] | 1.00 [0.81:1.20] | 0.31 [0.04:0.51] | 316 |
|  | 8 | 5.92e-3 [1.32e-3:0.27] |  | 0.78 [0.55:1.06] | 0.92 [0.69:1.13] | 4.80e-3[1.25e-3:0.25] | 171 |
|  | 9-15 | 0.84 [0.40:1.44] | 1.54e-3 [2.62^e^-4:0.31] | 2.09 [1.59 :2.57] | 2.78 [2.34 :3.63] | 0.28 [0.15:0.42] | 128 |
|  | all | 0.29 [0.22:0.41] | 0.28 [0.20:0.34] | 0.82 [0.79:0.86] | 1.40 [1.36:1.49] | 0.22 [0.16:0.28] | 2468 |
| Number of egg | 1 | 0.10 [6e-4:0.19] |  | 0.23 [0.13:0.32] | 0.33 [0.27:0.38] | 0.20 [0.01:0.35] | 829 |
|  | 2 | 0.13 [0.08:0.17] |  | 0.27 [0.23:0.32] | 0.40 [0.36:0.44] | 0.23 [0.14:0.31] | 2035 |
|  | 3 | 0.33 [0.23:0.44] |  | 0.31 [0.24:0.39] | 0.64 [0.57:0.72] | 0.43 [0.32:0.54] | 1912 |
|  | 4 | 0.26 [0.16:0.34] |  | 0.41 [0.32:0.48] | 0.65 [0.59:0.73] | 0.32 [0.22:0.42] | 1738 |
|  | 5 | 0.33 [0.21:0.50] |  | 0.34 [0.24:0.45] | 0.69 [0.61:0.80] | 0.44 [0.28:0.58] | 1424 |
|  | 6 | 0.38 [0.17:0.55] |  | 0.37 [0.25:0.54] | 0.72 [0.61:0.87] | 0.39 [0.23:0.60] | 816 |
|  | 7 | 0.37 [0.18:0.69] |  | 0.24 [0.12:0.46] | 0.70 [0.61:0.92] | 0.57 [0.29:0.77] | 523 |
|  | 8 | 0.40 [0.22:0.94] |  | 0.37 [0.16:0.65] | 0.89 [0.69:1.18] | 0.54 [0.27:0.78] | 204 |
|  | 9-15 | 1.06 [0.54:1.41] | 2.77^e-^3[2.69^e^-4:0.21] | 0.38 [0.28 :0.50] | 1.46 [1.10 :1.79] | 0.68[0.42:0.76] | 224 |
|  | all | 0.34 [0.29:0.41] | 0.04 [0.01:0.07] | 0.24 [0.23:0.26] | 0.64 [0.60:0.68] | 0.55 [0.49:0.61] | 3013 |
| Sperm viability | 1 | 7.93e-5  [6.64e-9:1.22e-2] | 1.30e-2  [4.68e-3:2.17e-02] | 9.97e-3  [8.36e-03:1.18e-2] | 2.69e-2  [2.25e-2:3.38e-2] | 3.12e-3  [2.02e-7:4.12e-1] | 127 |
|  | 2 | 6.81e-6  [2.34e-10:1.93e-3] | 1.17e-2  [1.00e-2:1.41e-2] | 6.84e-3  [6.40e-03:7.65e-3] | 2.00e-2  [1.78e-2:2.16e-2] | 5.49e-4  [1.27e-8:9.53e-2] | 587 |
|  | 3 | 3.02e-5  [1.10e-06:3.59e-3] | 9.28e-3  [7.36e-3:1.22e-2] | 7.14e-3  [6.70e-03:8.13e-3] | 1.86e-2  [1.67e-2:2.05e-2] | 1.92e-3  [5.53e-5:1.89e-1] | 519 |
|  | 4 | 3.79e-3  [4.61e-04:7.19e-3] | 8.67e-3  [6.01e-3:1.21e-2] | 1.01e-2  [9.03e-03:1.11e-2] | 2.36e-2  [2.05e-2:2.56e-2] | 1.74e-1  [3.76e-2:3.14e-1] | 400 |
|  | 5 | 8.04e-6  [1.49e-10 :2.50e-3] | 1.16e-2  [8.40e-3:1.46e-2] | 1.21e-2  [1.07e-02:1.38e-2] | 2.50e-2  [2.13e-2:2.73e-2] | 5.60e-4  [6.46e-9:1.02e-1] | 306 |
|  | 6 | 4.16e-5  [6.08e-9 :6.60e-3] | 8.99e-3  [3.86e-3:1.30e-2] | 9.77e-3  [8.40e-03:1.16e-2] | 2.20e-2  [1.77e-2:2.52e-2] | 1.61e-3  [3.43e-7:2.94e-1] | 165 |
|  | 7 | 4.93e-5 [3.70e-10 :7.66e-3] | 7.28e-3  [2.50e-3:1.25e-2] | 1.05e-2  [8.99e-03:1.33e-2] | 2.09e-2  [1.72e-2:2.58e-2] | 2.42e-3  [1.76e-8:3.32e-1] | 116 |
|  | 8 | 9.43e-5 [6.99e-8:1.15e-2] | 6.95e-5 [1.03e-91.21e-2] | 1.22e-2  [9.29e-03:1.58e-2] | 2.17e-2  [1.68e-2:2.83e-2] | 3.72e-3  [2.53e-6:4.60e-1] | 74 |
|  | 9-15 | 9.90e-6 [5.49e-10 :3.02e-3] | 8.96e-3[5.43e-3:1.16e-2] | 1.18e-2  [1.02e-02:1.33e-2] | 2.05e-2  [1.80e-2:22.72e-2] | 5.19e-4  [2.45e-8:1.32e-1] | 92 |
|  | all | 6.53e-4  [1.94e-5:1.68e-3] | 7.72e-3  [6.64e-3:8.95e-3] | 1.39e-2  [1.35e-2:1.45e-2] | 2.21e-2  [2.14e-2:2.38e-2] | 2.60e-2  [4.63e-6:7.31e-2] | 1144 |

The different variance components are: Va: the additive genetic variance, Vpe: the permanent environment variance, Vr: the residual variance. The phenotypic variances (Vp) were post calculated on model estimates. Poisson-heritability (h^2^) was calculated as

H^2^=Va/{Va+Vpe+Vr+log[1/exp(Xage)+1]}, where (Xage) is the predicted trait value given speciﬁc ﬁxed age (Nakagawa and Schielzeth 2010; Reid et al. 2011). Nind represents the number of individuals collected for each age class. Posterior mode (PM) and 95% confidence interval (95% CI) of estimates are provided. All the variance estimates are given on the latent scale and not back-transformed to the phenotypic scale.

Nakagawa, S., and H. Schielzeth. (2010). Repeatability for Gaussian and non-Gaussian data: a practical guide for biologists. Biological Reviews, 85:935–956.

Reid, J. M., P. Arcese, R. J. Sardell, and L. F. Keller. (2011). Additive genetic variance, heritability, and inbreeding depression in male extra-pair reproductive success.,” American Naturalist,177:177–187.

Table S1B: Fixed effect estimates from univariate models.

| **Univariate animal models models with age classes** | | | | | **Animal model with all data** | | |  |  |
| --- | --- | --- | --- | --- | --- | --- | --- | --- | --- |
| **Ejaculate size** | **Age** | **Age classes** | **Intercept** | **Dsle (x10^-2^)** | **Doc (x10^-3^)** | **Age** | **Dsle (x10^-2^)** | **Doc (x10^-3^)** | **Age** |
|  | **1** | **1** | 0.59 [0.25:0.96] | 1.68 [1.07:2.76] | 7.94 [6.76:9.91] |  | 1.82 [1.64 :1.96] | 1.45 [1.31 :1.59] | 0.65 [0.44:0.77] |
|  | **2** | **2** | 1.35 [1.11:1.59] | 2.85 [2.48:3.17] | 1.35 [0.93:1.61] |  |  |  | 0.95 [0.9:0.98] |
|  | **3** | **3** | 1.60 [1.38:1.85] | 3.44 [3.10:3.77] | NE |  |  |  | 1.01 [0.99:1.06] |
|  | **4** | **4** | 1.70 [1.53:1.97] | 3.31 [2.96:3.70] | NE |  |  |  | 1.13 [1.1:1.17] |
|  | **5** | **5** | 1.74 [1.49:1.95] | 3.63 [3.25:4.04] | 1.05 [0.91:1.46] |  |  |  | 1.19 [1.12:1.19] |
|  | **6** | **6** | 2.05 [1.82:2.27] | 1.99 [1.48:2.48] | 0.95 [0.56:1.32] |  |  |  | 1.03 [1:1.08] |
|  | **7** | **7** | 2.14 [1.82:2.41] | 3.51 [2.81:3.94] | NE |  |  |  | 1.04 [0.99:1.08] |
|  | **8** | **8-15** | 2.62 [2.33:2.84] | 1.49 [1.08:2.00] | 1.80 [1.39:2.14] |  |  |  | 0.94 [0.89:0.98] |
|  | **9** |  |  |  |  | -0.12 [-0.16:-0.08] |  |  | NE |
|  | **10** |  |  |  |  | -0.32 [-0.37:-0.25] |  |  | 0.64 [0.56:0.70] |
|  | **11** |  |  |  |  | -0.40 [-0.49:-0.34] |  |  | 0.56 [0.48:0.66] |
|  | **12** |  |  |  |  | -0.30 [-0.37:-0.19] |  |  | 0.68 [0.57:0.78] |
|  | **13** |  |  |  |  | -0.30 [-0.44:-0.18] |  |  | 0.68 [0.51:0.78] |
|  | **14** |  |  |  |  | -0.28 [-0.47:-0.12] |  |  | 0.62 [0.44:0.84] |
|  | **15** |  |  |  |  | -0.24 [-0.43:-0.09] |  |  | 0.71 [0.47:0.87] |
| **Sexual display effort** | **1** | **1** | 1.93 [1.77:2.05] |  |  |  |  |  | 1.56 [1.41:1.66] |
|  | **2** | **2** | 2.88 [2.77:3.02] |  |  |  |  |  | 1.15 [1.06:1.20] |
|  | **3** | **3** | 3.03 [2.82:3.17] |  |  |  |  |  | 1.56 [1.51:1.65] |
|  | **4** | **4** | 3.39 [3.29:3.59] |  |  |  |  |  | 1.75 [1.65:1.80] |
|  | **5** | **5** | 3.57 [3.38:3.76] |  |  |  |  |  | 1.70 [1.60:1.78] |
|  | **6** | **6** | 3.76 [3.58:3.92] |  |  |  |  |  | 1.77 [1.68:1.87] |
|  | **7** | **7** | 3.92 [3.73:4.11] |  |  |  |  |  | 1.71 [1.61:1.86] |
|  | **8** | **8** | 4.05[3.84:4.26] |  |  |  |  |  | 1.71 [1.54:1.86] |
|  | **9** | **9-15** | 3.06[2.61:3.57] |  |  |  |  |  | 1.40 [1.19:1.56] |
|  | **10** |  |  |  |  | 0.28[-0.24:0.78] |  |  | 1.71 [1.51:2.04] |
|  | **11** |  |  |  |  | -0.59[-1.14:0.04] |  |  | 1.01 [0.81:1.44] |
|  | **12** |  |  |  |  | -0.36[-0.97:0.22] |  |  | 1.45 [0.95:1.61] |
|  | **13** |  |  |  |  | 0.08[-0.62:0.85] |  |  | 1.70 [1.31:2.13] |
|  | **14** |  |  |  |  | -0.25[-1.15:0.68] |  |  | 1.56 [1.04:2.16] |
|  | **15** |  |  |  |  | -0.98[-1.90:0.05] |  |  | 1.03 [0.22:1.48] |
| **Number of egg** | **1** | **1** | 1.33 [1.18:1.42] |  |  |  |  |  | 0.58 [0.46:0.69] |
|  | **2** | **2** | 1.55 [1.45:1.66] |  |  |  |  |  | 0.55 [0.48:0.60] |
|  | **3** | **3** | 1.38 [1.26:1.55] |  |  |  |  |  | 0.70 [0.62:0.74] |
|  | **4** | **4** | 1.46 [1.34:1.61] |  |  |  |  |  | 0.79 [0.72:0.84] |
|  | **5** | **5** | 1.54 [1.38:1.68] |  |  |  |  |  | 0.93 [0.87:1.00] |
|  | **6** | **6** | 1.61 [1.42:1.79] |  |  |  |  |  | 1.06 [0.99:1.13] |
|  | **7** | **7** | 1.66 [1.43:1.85] |  |  |  |  |  | 1.13 [1.04:1.20] |
|  | **8** | **8** | 1.55[1.30:1.83] |  |  |  |  |  | 1.17 [1.06:1.25] |
|  | **9** | **9-15** | 1.00[0.66:1.36] |  |  |  |  |  | 1.18 [1.07:1.29] |
|  | **10** |  |  |  |  | 0.19[-0.01:0.39] |  |  | 1.23 [1.09:1.36] |
|  | **11** |  |  |  |  | 0.15[-0.08:0.37] |  |  | 1.17 [1.01:1.33] |
|  | **12** |  |  |  |  | 0.26[0.04:0.49] |  |  | 1.19 [1.10:1.43] |
|  | **13** |  |  |  |  | 0.23[-0.02:0.53] |  |  | 1.21 [0.95:1.38] |
|  | **14** |  |  |  |  | 0.08[-0.27:0.45] |  |  | 1.05 [0.81:1.35] |
|  | **15** |  |  |  |  | -0.02[-0.38:0.35] |  |  | 1.01 [0.68:1.26] |
| **Sperm vialbility** | **1** | **1** | 1.00[0.97:1.05] |  |  |  |  |  | 0.99 [0.96:1.01] |
|  | **2** | **2** | 1.06[1.05:1.07] |  |  |  |  |  | 0.06 [0.04:0.09] |
|  | **3** | **3** | 1.08[1.06:1.10] |  |  |  |  |  | 0.10 [0.07:0.12] |
|  | **4** | **4** | 1.03[1.00:1.06] |  |  |  |  |  | 0.06 [0.04:0.08] |
|  | **5** | **5** | 1.05[1.04:1.08] |  |  |  |  |  | 0.06 [0.03:0.08] |
|  | **6** | **6** | 1.07[1.05:1.10] |  |  |  |  |  | 0.07 [0.05:0.10] |
|  | **7** | **7** | 1.06[1.03:1.09] |  |  |  |  |  | 0.06 [0.03:0.09] |
|  | **8** | **8-15** | 0.76 [0.72:0.79] |  |  |  |  |  | 0.07 [0.04:0.11] |
|  | **9** |  |  |  |  | -0.06 [-0.13:-0.02] |  |  | -0.02 [-0.06:0.03] |
|  | **10** |  |  |  |  | -0.02 [-0.10:0.07] |  |  | 0.10 [-0.01:0.15] |
|  | **11** |  |  |  |  | -0.04 [-0.15:0.05] |  |  | 0.05 [-0.03:0.15] |
|  | **12** |  |  |  |  | -0.13 [-0.22:-0.03] |  |  | -0.04 [-0.11:0.06] |
|  | **13** |  |  |  |  | -0.20 [-0.31:-0.08] |  |  | -0.16 [-0.25:-0.05] |
|  | **14** |  |  |  |  | -0.19 [-0.30:-0.09] |  |  | -0.16 [-0.25:-0.05] |
|  | **15** |  |  |  |  | -0.02 [-0.14:0.08] |  |  | 0.03 [-0.08:0.13] |

Age correction was fitted as categorical fixed effect in 8-15 age classes univariate models and for the global animal model. For ejaculate size univariate animal models the date since the last ejaculation (Dsle) and the date of collect (Doc) were fitted as fixed effects. NE were values not estimated by the model.
