# Supplementary material for: Quantitative Genetics of the Aging of Reproductive Traits in the Houbara Bustard
Source: PLoS One. 2015 Jul 28;10(7):e0133140. doi: 10.1371/journal.pone.0133140 (PMC4517785; doi:10.1371/journal.pone.0133140)
Supplement: S2 Table — (DOCX) [file pone.0133140.s004.docx]

Table S2A: Estimates of additive genetic and permanent environment polynomial function from random regression animal models estimated with MCMCglmm package and Poisson family.

| **Random regression models** | **Ejaculate size** | | **Courtship display rate** | | **Egg production** | |
| --- | --- | --- | --- | --- | --- | --- |
|  | **Va** | **Vpe** | **Va** | **Vpe** | **Va** | **Vpe** |
| **Intercept** | 0.64 [0.40:0.72] | 39.36 [35.27:50.47] | 0.79 [0.26:1.88] | 0.94 [0.32:2.01] | 1.18 [0.95:1.63] | 0.29 [0.06:0.58] |
| **Age** | -- | 46.37 [42.04:59.78] | 0.86 [0.19:2.79] | 1.63 [0.66:3.51] | 0.17 [0.07:0.34] | 0.84 [0.49:1.37] |
| **Age2** | -- | 12.44 [10.15:13.77] | 0.34 [0.19:0.86] | 0.88 [0.49:1.35] | -- | 0.28 [0.16:0.37] |
| **cov(Int,age)** | -- | 42.18 [37.88:54.46] | 0.26 [-0.09:1.76] | 0.82 [0.09:2.18] | 0.39 [0.25:0.67] | 0.55 [0.15:0.83] |
| **cov(Int,age2)** | -- | 22.32 [18.44:26.06] | -0.01 [-0.25:0.68] | 0.47 [2.84e-3:1.23] | -- | 0.21 [0.08:0.37] |
| **cov(age,age2)** | -- | 23.20 [19.99:27.85] | 0.42 [0.08:1.33] | 1.04 [0.44:2.02] | -- | 0.4 [0.27:0.67] |

We partitioned individual variation across age into additive genetic variance (Va) andpermanent environment (Vpe) using a selection strategy developed in material and method (with Intercept, Age (or slope) and Age2 (slope2) and the respective covariance between them (noted cov(,)). Posterior mode (PM) and 95% credible interval (95% CI) of estimates are provided. Estimates of fixed parameters and residuals are provided in Table S3.

Table S2A: Fixed effect and residual variance (Vr) estimates from random regression animal models estimated with MCMCglmm package and Poisson family. Age correction was fitted as categorical fixed effect into models. A residual variance was fitted for each age. For ejaculate size univariate animal models the date since the last ejaculation (Dsle) and the date of collect (Doc) were fitted as fixed effects.

|  | **Ejaculate size** | **Sexual display effort** | **Egg production** |
| --- | --- | --- | --- |
| **Intercept** | -0.33 [-0.52:-0.13] | 1.50 [1.35:1.67] | 0.87 [0.76:0.99] |
| **Age:2** | 1.69 [1.60:1.78] | 1.16 [1.07:1.28] | 0.44 [0.37:0.51] |
| **Age:3** | 1.92 [1.80:2.04] | 1.64 [1.48:1.78] | 0.51 [0.41:0.58] |
| **Age:4** | 2.04 [1.91:2.19] | 1.81 [1.61:1.99] | 0.55 [0.43:0.64] |
| **Age:5** | 2.04 [1.93:2.21] | 1.81 [1.58:2.01] | 0.65 [0.55:0.78] |
| **Age:6** | 1.96 [1.79:2.07] | 1.95 [1.71:2.19] | 0.77 [0.60:0.88] |
| **Age:7** | 2.04 [1.81:2.10] | 1.96 [1.74:2.26] | 0.85 [0.65:0.98] |
| **Age:8** | 2.17 [2.02:2.38] | 2.11 [1.84:2.41] | 0.83 [0.63:1.02] |
| **Age:9** | 2.25 [1.98:2.49] | 1.89 [1.53:2.23] | 0.84 [0.54:1.01] |
| **Age:10** | 2.31 [1.98:2.70] | 2.35 [1.89:2.80] | 0.88 [0.58:1.15] |
| **Age:11** | 3.11 [2.56:3.57] | 1.52 [0.61:2.24] | 0.65 [0.35:1.04] |
| **Age:12** | 3.99 [3.14:4.58] | 1.90 [1.05:2.79] | 0.69 [0.35:1.09] |
| **Age:13** | 5.14 [4.21:6.06] | 2.84 [1.45:3.74] | 0.69 [0.13:1.20] |
| **Age:14** | 5.82 [4.72:7.22] | 1.76 [0.12:3.16] | 0.62 [-0.21:1.23] |
| **Age:15** | 7.05 [4.99:7.91] | 0.54 [-1.21:2.66] | 0.18 [-0.43:1.17] |
| **Doc (x10^-4^)** | 7.91 [6.26:8.85] | -- | -- |
| **Dsle(x10^-2^)** | 2.54 [2.43:2.74] | -- | -- |
| **Vr.1** | 0.91 [0.85:0.96] | 0.71 [0.57:0.81] | 0.24 [0.19:0.28] |
| **Vr.2** | 0.47 [0.46:0.48] | 0.71 [0.63:0.77] | 0.23 [0.19:0.26] |
| **Vr.3** | 0.45 [0.44:0.46] | 0.55 [0.48:0.61] | 0.26 [0.22:0.30] |
| **Vr.4** | 0.40 [0.40:0.43] | 0.45 [0.39:0.52] | 0.25 [0.21:0.29] |
| **Vr.5** | 0.38 [0.37:0.39] | 0.56 [0.47:0.65] | 0.24 [0.19:0.27] |
| **Vr.6** | 0.43 [0.41:0.45] | 0.51 [0.40:0.58] | 0.23 [0.19:0.29] |
| **Vr.7** | 0.36 [0.35:0.38] | 0.53 [0.43:0.67] | 0.24 [0.19:0.30] |
| **Vr.8** | 0.27 [0.26:0.29] | 0.43 [0.31:0.57] | 0.43 [0.32:0.55] |
| **Vr.9** | 0.31 [0.28:0.33] | 0.77 [0.50:1.11] | 0.60 [0.42:0.84] |
| **Vr.10** | 0.41 [0.34:0.45] | 0.69 [0.41:1.06] | 0.47 [0.29:0.63] |
| **Vr.11** | 0.39 [0.30:0.44] | 3.42 [1.78:5.39] | 0.61 [0.38:0.88] |
| **Vr.12** | 0.45 [0.38:0.57] | 2.54 [1.36:4.31] | 0.56 [0.37:0.86] |
| **Vr.13** | 0.36 [0.32:0.46] | 1.51 [0.65:2.75] | 0.74 [0.39:1.17] |
| **Vr.14** | 0.34 [0.28:0.45] | 0.94 [0.48:2.25] | 0.80 [0.44:1.46] |
| **Vr.15** | 0.42 [0.37:0.59] | 1.02 [0.51:2.17] | 0.63 [0.37:1.27] |
