# Supplementary material for: Quantitative Genetics of the Aging of Reproductive Traits in the Houbara Bustard
Source: PLoS One. 2015 Jul 28;10(7):e0133140. doi: 10.1371/journal.pone.0133140 (PMC4517785; doi:10.1371/journal.pone.0133140)
Supplement: S1 Text — (DOCX) [file pone.0133140.s005.docx]

**Text S1: The number of individuals per age for the traits under study, information on relatedness and power analyses on univariate age-classes**

1. The number of individuals per age for the traits under study.

| **Age** | **sexual display effort** | **sperm viability** | **number of egg produced** | **ejaculate size** |
| --- | --- | --- | --- | --- |
| 1 | 1638 | 128 | 829 | 486 |
| 2 | 2035 | 586 | 2035 | 1175 |
| 3 | 1450 | 519 | 1912 | 1004 |
| 4 | 1080 | 400 | 1738 | 766 |
| 5 | 887 | 306 | 1424 | 691 |
| 6 | 552 | 165 | 816 | 431 |
| 7 | 316 | 116 | 523 | 279 |
| 8 | 171 | 74 | 310 | 149 |
| 9 | 111 | 32 | 204 | 93 |
| 10 | 56 | 10 | 118 | 32 |
| 11 | 45 | 7 | 90 | 20 |
| 12 | 41 | 8 | 78 | 15 |
| 13 | 23 | 6 | 49 | 12 |
| 14 | 13 | 6 | 31 | 9 |
| 15 | 12 | 6 | 28 | 7 |
| 16 | 7 | 5 | 23 | 6 |
| 17 | 5 | 4 | 10 | 5 |
| 18 | 5 | 5 | 8 | 5 |
| 19 | 3 | - | 7 | 3 |
| 20 | 3 | 3 | 7 | 3 |
| 21 | 3 | 3 | 5 | 3 |
| 22 | 3 | 1 | 4 | 3 |
| 23 | 1 | - | 2 | - |

1. Information on relatedness.

Relatedness is calculated between all pairs of individuals, here a pair is any dyad of individuals from the population. Because of the large number of individuals, the number of pairs is reported on the logarithmic scale.


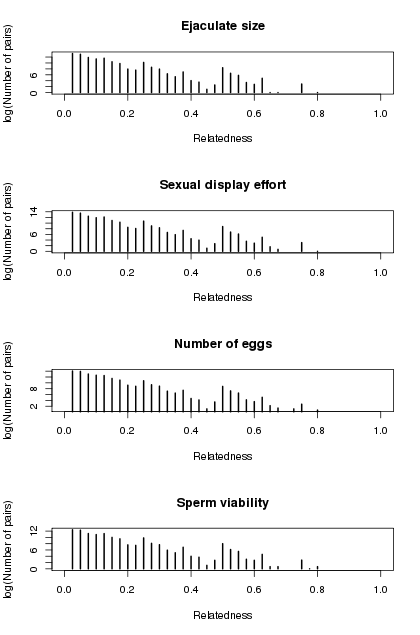


1. Relatedness by age-class for ejaculate size, courtship display rate, egg production and sperm viability.

| Relatedness | Ejaculate size | | Courtship display rate | | Egg production | | Sperm viability | |
| --- | --- | --- | --- | --- | --- | --- | --- | --- |
| Age | 0.25 | 0.50 | 0.25 | 0.50 | 0.25 | 0.50 | 0.25 | 0.50 |
| 1 | 10189 | 1945 | 28403 | 1945 | 14484 | 2635 | 3135 | 770 |
| 2 | 19289 | 3292 | 34726 | 3292 | 30064 | 4619 | 10632 | 1907 |
| 3 | 15967 | 2689 | 23763 | 2689 | 28541 | 4166 | 9495 | 1562 |
| 4 | 12200 | 2067 | 17820 | 2067 | 27075 | 3736 | 6970 | 1200 |
| 5 | 10773 | 1858 | 13741 | 1858 | 23030 | 3176 | 5134 | 947 |
| 6 | 6424 | 1191 | 2517 | 1191 | 13580 | 1910 | 2319 | 565 |
| 7 | 3885 | 766 | 4483 | 766 | 8779 | 1258 | 3885 | 766 |
| 8 | 2291 | 468 | 2146 | 444 | 4704 | 736 | 706 | 241 |
| 9-15 |  |  | 1201 | 286 | 2587 | 487 | 252 | 108 |

Pairs having relatedness of 0.5 (parent-offspring or fullsibs) and pairs having relatedness of 0.25 (half sibs, nieces or nephews, grandparents / grandchildren)

1. Method and results of the simulation tests that we used to assess the ability of our data to estimate Va

In order to assess whether the patterns of additive genetic variance would result from an overestimation of additive genetic variance in the older age classes (e.g. difficulty to separate additive genetic and permanent environment effects), we run some simulation tests. In these analyses, we simulated 50 phenotypic data sets for several age-class based on (i) the number of observations per individual per age-class and,(ii) the pedigree corresponding to each age class and (iii) a Va equal to 0.5 for sperm traits and 1 for other traits (Morrissey and Wilson 2010). For each trait, we simulated data sets for four age classes: (1) seven years old, (2) 8 years old, (3) 8 years and older until 16 years old and (4) 9 years and older. For each age-class, the 50 simulated datasets differed in the breeding values associated with individuals. The simulations of dataset were done using pedantic ([Morrissey and Wilson 2010](#_ENREF_1)).

Then, we run univariate animal models (see material and methods) on these simulated datasets. If no bias exists due to the pedigree structure, the estimates of Va should include the value of Va used for simulating the data.

See the results below.

Morrissey, M. B., and A. J. Wilson. 2010. PEDANTICS: an R package for pedigree-based genetic simulation and pedigree manipulation, characterization and viewing. Molecular Ecology **10**:711-719.

Additive genetic variance (Va) estimated for the **ejaculate size** trait using the four different age classes defined above.


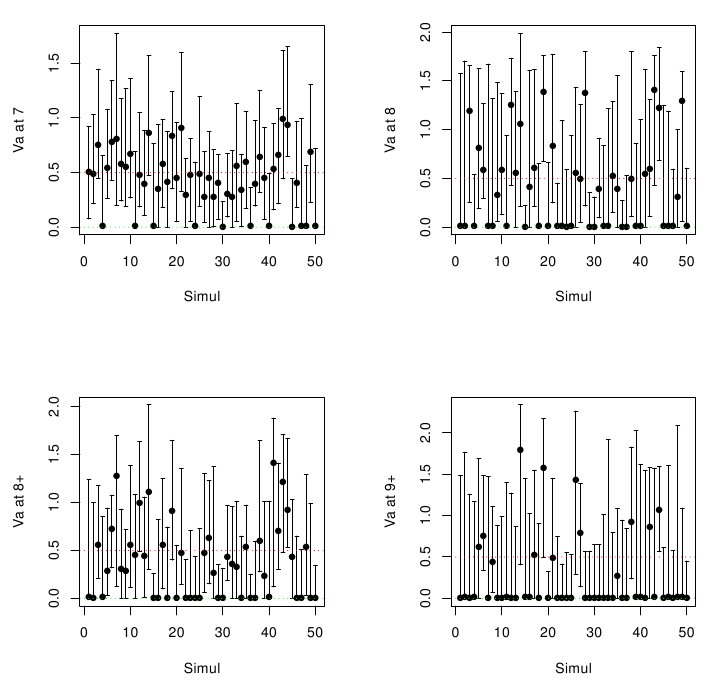


The posterior estimates of Va on the 50 simulated datasets show that age 8+ provided better estimate than age 9+, as the models tend to collapse on 0 more frequently in this age class. When Va is estimated, it is not significantly different from the initial value of 0.5. No systematic overestimation in age classes 8+ or 9+ was detected.

Additive genetic variance (Va) estimated for the **courtship display rate** trait using the four different age classes defined above.


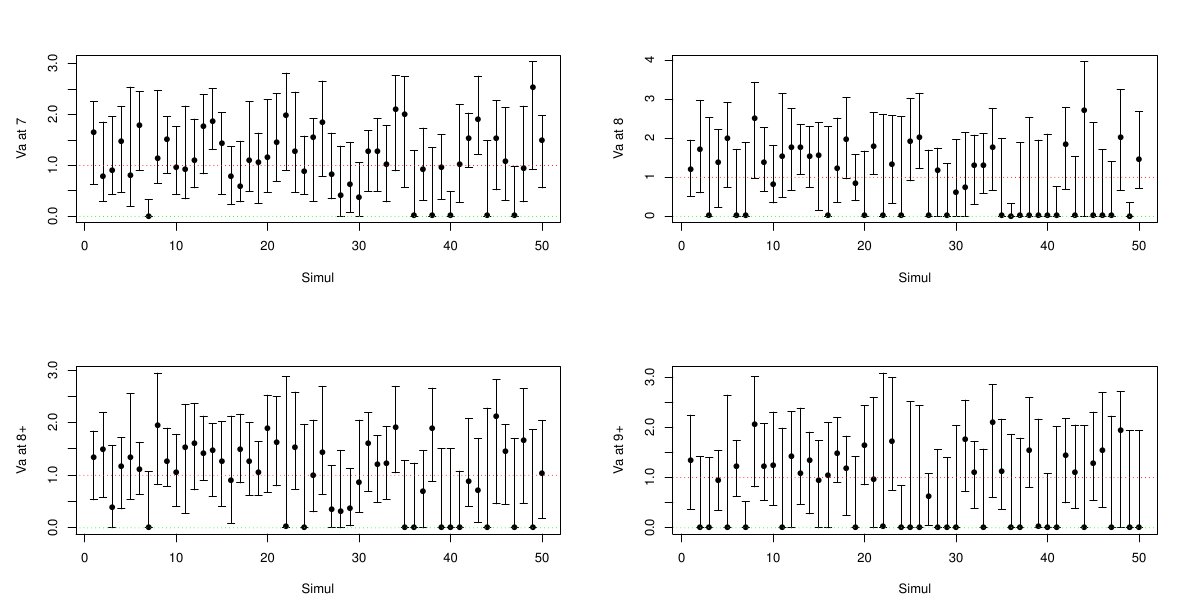


The posterior estimates of Va on the 50 simulated datasets show a good ability to predict simulated Va although at age 8, Va may be slightly underestimated, which may explain our results of low Va at age 8. In the age class 8+ and 9+, no overestimation of Va was detected: either the model collapsed on 0 or estimated Va accurately.

Additive genetic variance (Va) estimated for the **egg production** trait using the four different age classes defined above.


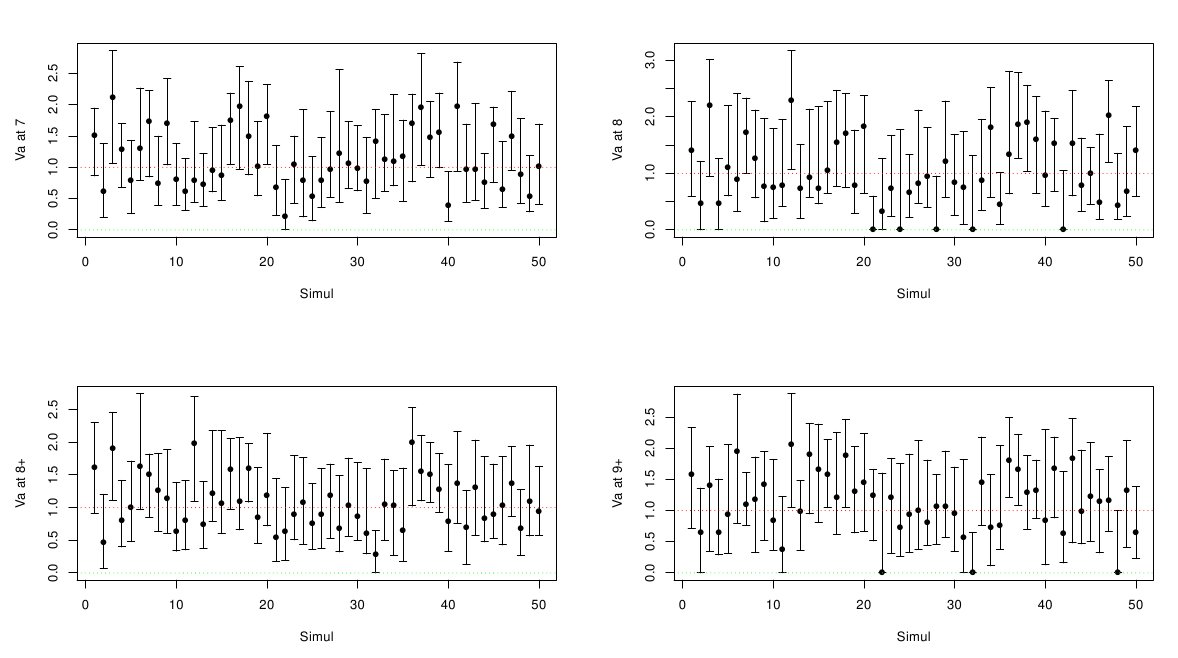


The posterior estimates of Va on the 50 simulated datasets show a good ability to predict simulated Va for all age-classes. In the age class 9+, no overestimation of Va was detected, so that increased Va in our main results is not due to an underlying bias.

Additive genetic variance (Va) estimated for **sperm viability** trait using the four different age classes defined above.


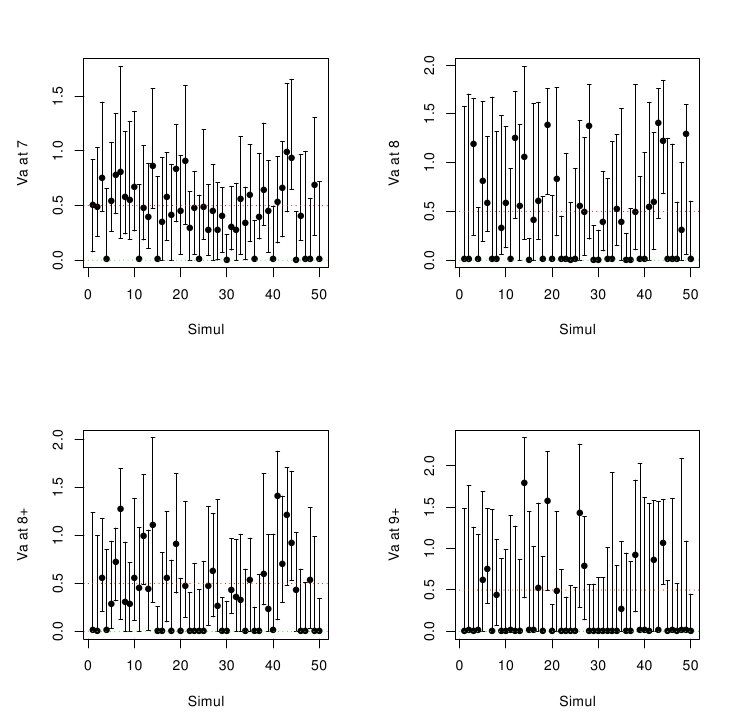


The posterior estimates of Va on the 50 simulated datasets show that age 8+ provided better estimates than estimates that could be obtained from data restricted to age class 9+. Note that even at age 8+, Va may be slightly underestimated. Results for age 7 suggest that the null Va obtained in the result is not due to power issues.
