# Supplementary material for: Quantitative Genetics of the Aging of Reproductive Traits in the Houbara Bustard
Source: PLoS One. 2015 Jul 28;10(7):e0133140. doi: 10.1371/journal.pone.0133140 (PMC4517785; doi:10.1371/journal.pone.0133140)
Supplement: S2 Text — (DOCX) [file pone.0133140.s006.docx]

**Text S2: Model parameterisations and post-test methodologies for univariate and bivariate animal models**

1. Approach used for bivariate models between the older age-class and the other age-classes

For sexual display effort and number of eggs, in the eight first age classes, the permanent environment effect (**pe**) is not modelled as only one observation per individual is defined. The presence of **pe** for the older age class requires using a slightly different modelling approach of the residual variance-covariance matrix in the bivariate models. When only one observation per individual is available, the residual variance represent an inter-individual variance, whereas when several measurements per individual are performed, the inter-individual variance is included in **pe**, and the residual variance represent then the intra-individual variance. To reflect this in the models, the residual variance of the seven first age classes dependent variable were fixed to 0.001 (fixing to 0 is not allowed by the software) to force residual information going into the permanent environment part of model. Consequently, the residual covariance between one of the seven first age classes and the 8-15 age class was also set to 0. See <http://www.wildanimalmodels.org/tiki-index.php?page=cross-age+correlation> for more detailed information.

1. Age-classes pair-wise post-tests methodologies

Following univariate models, we used a post hoc tests based on the posterior distributions of additive genetic variance for each age class.

Test for the pair-wise difference: We computed pair-wise comparisons to assess the differences in Va estimates between the age-classes. To assess the differences between two age-classes, we took advantage of the full posterior distribution given by the Bayesian approach and subtracted the posterior distribution of Va in a given age-class from the posterior distribution of Va in another age-class. This gave 1000 independent estimates of the difference in Va between two age-classes. The significance of this difference was tested by the number of times the difference was positive or negative. This comparison was done among all age-classes and significant difference in Va between two age-classes is represented by different letters in Fig. 3

For the bivariate models, the significance of genetic correlations was simply assessed by whether the 95% confidence interval overlapped zero.
