# Supplementary material for: Quantitative Genetics of the Aging of Reproductive Traits in the Houbara Bustard
Source: PLoS One. 2015 Jul 28;10(7):e0133140. doi: 10.1371/journal.pone.0133140 (PMC4517785; doi:10.1371/journal.pone.0133140)

**Text S3: Details about the random regression animal models**: the deviance information criterion (DIC), back-transformation of the Legendre polynomial function into Va and age-related variation of the additive genetic variance plotted from the best random regression animal model found in Table 3-5 using ASReml software.

1. The problems of using DIC for Poisson random regression animal models.

Assessing the significance of effects in a Bayesian context is difficult as it is not the main philosophy of the analysis that relies on the 95% confidence interval. The Deviance Information Criterion (DIC) has been developed for model comparison. DIC is based on the estimate of the likelihood of a model penalizing by its complexity (Spiegelhalter et al. 2002). Nevertheless, for model comparison with DIC, likelihood estimate is required to be `focused' at the right level. In fact, DIC can be computed at different level: the conditional DIC uses likelihood conditioned on parameters at the observation level, whereas the group- and top-level DICs use partially marginalized likelihoods (Millar 2009). Although, for scientific inference the top-level DICs should be used, conditional DIC is easier to estimate from a computational perspective. Consequently, most software (as MCMCglmm package : Hadfield 2010) provides conditional DIC and is therefore not suitable for model comparison (Spiegelhalter et al. 2002, Millar 2009). As no reliable selection model criteria yet exists for MCMCglmm model using Poisson distributed data, we used a REML approach and followed the selection strategy as in Brommer et al. (2010) on the data previously log transformed (except for viability, Gaussian distribution)

Brommer, J. E., K. Rattiste, and A. J. Wilson. 2010. The rate of ageing in a long-lived bird is not heritable. Heredity 104:363–70.

Hadfield, J. D. 2010. MCMC Methods for Multi-Response Generalized Linear Mixed Models: The MCMCglmm R Package. Journal of Statistical Software 33:1–22.

Millar, R. B. 2009. Comparison of hierarchical Bayesian models for overdispersed count data using DIC and Bayes’ factors. Biometrics 65:962–9.

Spiegelhalter, D. J., N. G. Best, B. P. Carlin, and A. van der Linde. 2002. Bayesian measures of model complexity and fit. Journal of the Royal Statistical Society: Series B (Statistical Methodology) 64:583–639.

1. Transformation of Legendre polynomial function into an age specific variance-covariance matrix: the example of courtship display rate.

The estimates of the Legendre polynomial function *f(****a****, 2 ,* *St*age), which describe the additive genetic variation across ages for the courtship display effort trait, correspond to a 3x3 matrix of intercept, slope and slope² and their covariances (Q-matrix).


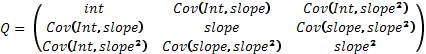


The age specific variance-covariance matrix of additive genetic variance (**G**) was obtained as G=zQz’ with z the vector of orthogonal polynomials evaluated at values of standardized age (from 1 to 15 years old). z’ was the tranposed matrix of z. We obtain a 15x15 G-matrix of variance-covariance matrix in which the diagonal were the additive genetic variance.

Our analyses were conducted in a Bayesian framework and 1000 independent estimates of Q matrix were provided. So, 1000 age specific variance-covariance matrix of additive genetic variance (**G**) were obtained and permitted to estimate posterior mode and 95% confidence interval of **G** matrices.

1. Age-related variation of the additive genetic variance plotted from the best random regression animal model found in Table 3-5 using ASReml software.


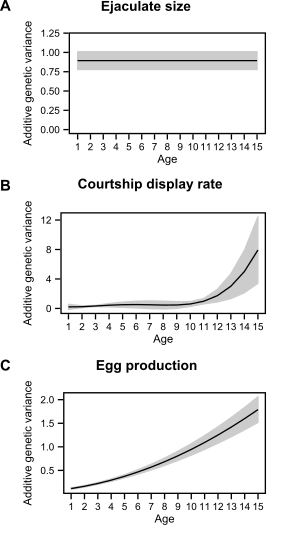

Supplement: S3 Text — The deviance information criterion (DIC), back-transformation of the Legendre polynomial function into Va and age-related variation of the additive genetic variance plotted from the best random regression animal model found in Tables 3–5 using ASReml software. (DOCX) [file pone.0133140.s007.docx]
